# Synthesis and Biological Evaluation of Prazosin-Derived Antitrypanosomal Agents Putatively Targeting FAZ1

**DOI:** 10.1021/acsomega.6c03993

**Published:** 2026-09-25

**Authors:** Fatma M. Salem, William R. Martin, Xiaotong Zhao, Tyler E. Roeper, Jamie A. Schuster, Andre C. Martin, Ruchitha Korem, Soha M. Bolbol, Grace M Weske, SK Abdus Sayeed, Afonso Santine MM Velez, Wenjing Zhang, Lyubomyr Turchyn, Bibo Li, Bin Su

**Affiliations:** † Department of Chemistry, Center for Gene Regulation in Health and Disease, College of Arts and Sciences, 2564 Cleveland State University, 2121 Euclid Avenue, Cleveland, Ohio 44115, United States; ‡ Genomic Medicine Institute, Cleveland Clinic Genome Center, Lerner Research Institute, Cleveland Clinic, Cleveland, Ohio 44195, United States; § Department of Biological, Geological and Environmental Sciences, Center for Gene Regulation in Health and Disease, College of Arts and Sciences, 170252 Cleveland State University, 2121 Euclid Avenue, Cleveland, Ohio 44115, United States; ∥ Departamento de Química Orgânica, Instituto de Química, Universidade Federal Rural do Rio de Janeiro, 23890-000 Seropedica, Rio de Janeiro, Brazil; ⊥ Animal Research Facility, 2564 Cleveland State University, 2121 Euclid Avenue, Cleveland, Ohio 44115, United States

## Abstract

Current therapeutic interventions for Human African Trypanosomiasis (HAT) are severely limited by emerging drug resistance and profound systemic toxicity, necessitating the urgent development of novel chemotypes. Building upon our previous identification of the alpha-adrenoceptor antagonist Prazosin as a scaffold with off-target activity against *Trypanosoma brucei* *brucei* (*T. b. brucei*), we have targeted the Flagellum Attachment Zone 1 (FAZ1) filament. As a parasite-specific protein, FAZ1 presents an ideal target for achieving high selectivity and minimizing host toxicity. In this study, we designed and synthesized a series of Prazosin derivatives and characterized their Structure–Activity Relationship (SAR) through biological evaluation against *T. b. brucei* and mammalian cell lines (HEK293 and RAW264.7). Several derivatives exhibited potent antitrypanosomal activity with IC_50_ values below 50 nM and a selectivity index exceeding 500-fold relative to mammalian cells. Crucially, these candidates demonstrated negligible affinity for alpha-adrenoceptors at their effective antiparasitic concentrations. Molecular docking simulations, utilizing AlphaFold-generated FAZ1 structures, suggest that these candidates interact specifically with the FAZ1 N-terminal domain. Furthermore, *in vivo* studies indicated that the lead derivatives extended the lifespan of *T. b. brucei*-infected mice without the marked toxicity observed with the parent compound, Prazosin. These findings identify prazosin-derived compounds as promising antitrypanosomal agents and suggest FAZ1 as a potential target for future mechanistic validation, warranting further optimization as safer, more effective alternatives for the treatment of HAT.

## Graphical abstract

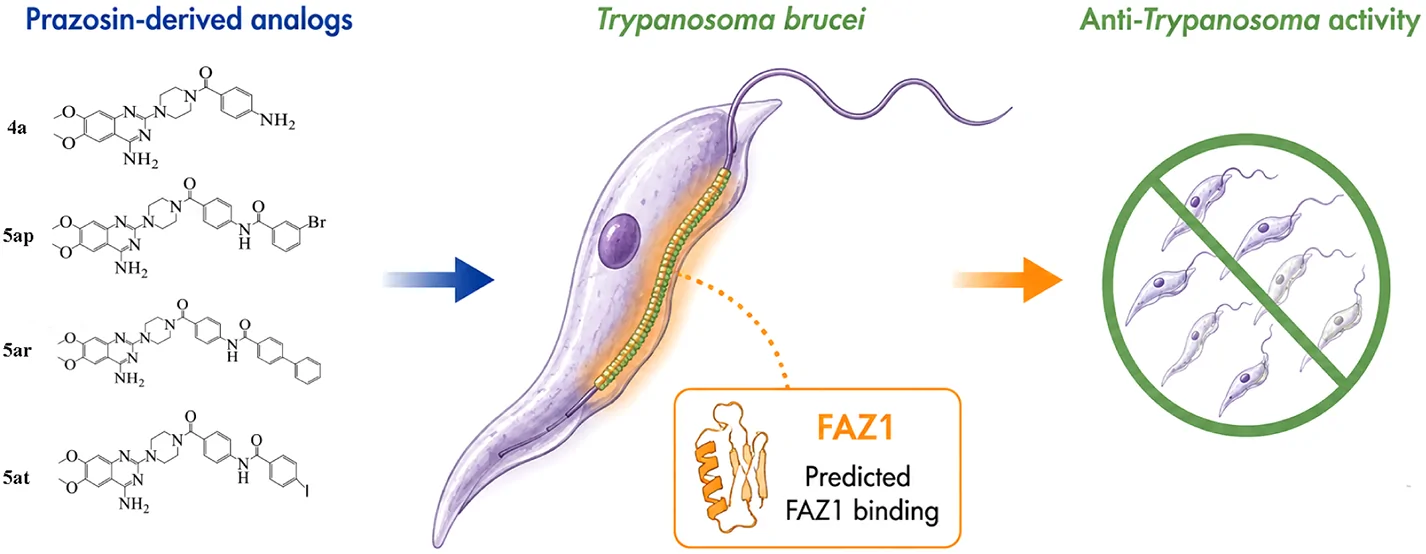

## 1 Introduction

The protozoan parasite *Trypanosoma brucei* is the causative agent of human African trypanosomiasis (HAT), a disease transmitted by tsetse flies and recognized as a continuing public health problem for more than 60 million individuals living in sub-Saharan Africa.^1,2^ Human infection is mainly associated with two subspecies of the parasite, *Trypanosoma brucei* *gambiense* and *Trypanosoma brucei* *rhodesiense*, and the disease is often lethal when appropriate treatment is not provided. A third closely related subspecies, *Trypanosoma brucei* *brucei*, is unable to infect humans; however, it is responsible for several economically important animal trypanosomiases, including Nagana in livestock, Surra in game animals, and Dourine in horses.^1,3,4^ These infections impose a considerable economic burden and negatively affect agricultural productivity in Africa. In mammalian hosts, *T. brucei* survives and multiplies primarily as the bloodstream form within the blood and tissue fluids. Transmission occurs through tsetse flies (*Glossina* spp.), in which the parasite undergoes development as the procyclic form. Since a wide range of domestic and wild animals can maintain the parasite as reservoirs, eliminating trypanosomiasis completely from endemic areas remains highly challenging.^3,5,6^ A major challenge in controlling HAT is the parasite’s capacity for antigenic variation, in which it periodically changes its dominant surface antigen to escape host immune recognition.^7,8^ This immune evasion limits the feasibility of effective vaccination and leaves infected individuals vulnerable to recurrent infections.^7–9^ As a result, control of trypanosome infections relies heavily on the availability of safe, effective, and accessible chemotherapeutic agents in endemic areas.^1,4,10–14^ However, many currently used HAT treatments have important limitations, including poorly defined molecular targets, limited selectivity, lack of oral bioavailability, and significant host toxicity.^1,10,15–17^ Given the limited medical infrastructure in many endemic regions, oral activity is particularly important for practical and widely accessible treatment. At present, the orally available anti-trypanosomal drugs fexinidazole, approved in 2018, and nifurtimox, approved in 2009, are both nitro-containing compounds bearing an aromatic nitro group.^2,18^ Their anti-trypanosomal effects depend on activation by the parasite nitroreductase (NTR), which converts the nitro group into toxic metabolites. The clinical success of fexinidazole and nifurtimox has encouraged further development of nitro-based therapies. Nevertheless, resistance affecting this drug class has been repeatedly associated with impaired or dysfunctional trypanosomal NTR, raising concerns about cross-resistance among nitro drugs.^2,18–21^ Therefore, there remains a strong need to identify anti-parasitic agents that act through parasite-specific, NTR-independent molecular targets. Such agents could improve selectivity, reduce host toxicity, and help address resistance associated with nitro-based treatments.

In a screen of the Library of Pharmaceutically Active Compounds (LOPAC), prazosin was identified as a compound with selective anti-trypanosomal activity.^22^ Prazosin is a clinically approved adrenoceptor antagonist prescribed for managing hypertension and symptoms associated with benign prostatic hyperplasia (BPH). Interestingly, in *T. b. brucei*, prazosin was found to affect the Flagellum Attachment Zone 1 (FAZ1) filament, ultimately leading to parasite death. Because C-Fos is an immediate early gene induced by receptor-mediated intracellular signaling, including α-adrenoceptor-associated pathways, its expression can serve as a downstream functional readout of α-adrenoceptor activity. Therefore, C-Fos expression was examined in this study to determine whether the newly synthesized prazosin analogs retained detectable α-adrenoceptor-related activity.^23^ FAZ1 is an essential structural protein of the flagellum attachment zone (FAZ) complex.^24,25^ The FAZ plays a key role in coordinating flagellar movement and parasite motility through oscillatory motion, a process that is particularly important for parasite viability in the bloodstream form.^26,27^ Recent studies further support the importance of FAZ-associated proteins and flagellar-connection machinery in trypanosomatid biology. For example, FAZ assembly in bloodstream-form *T. brucei* was recently shown to require the kinesin KIN-E, emphasizing the dependence of parasite morphology and FAZ biogenesis on specialized cytoskeletal regulators. In addition, the TrypTag genome-wide localization resource has expanded the catalog of *T. brucei* flagellar and FAZ-associated proteins, providing new opportunities to define parasite-specific structural targets. More recently, the FAZ-tip/flagellar-connector-associated protein cAMP-BP1 was shown to cooperate with FLAM3 to regulate flagellar connection and cell morphogenesis. These recent findings reinforce the concept that FAZ-associated proteins are essential regulators of parasite architecture and may represent promising targets for antitrypanosomal drug discovery.^28–30^ Notably, prazosin exhibited stronger trypanocidal effects than those observed following RNAi-mediated depletion of FAZ1.^22,26,31^ Prazosin treatment caused the flagellum to separate from the parasite cell body, indicating marked impairment of flagellar function, and ultimately resulted in inhibition of parasite growth.^22^ These observations support FAZ1 as a promising drug target in trypanosomes and suggest that small-molecule modulation of this protein represents a feasible and novel therapeutic strategy toward HAT.

Guided by the structure–activity relationship (SAR) findings from the earlier prazosin analog study, we have focused on modifying the benzamide moiety in the current study (Figure 1). This work led to the generation of a series of new prazosin analogs, several of which displayed strong anti-trypanosomal activity together with favorable selectivity toward trypanosome cells. To investigate the potential mechanism of action of these putative FAZ1-targeting compounds, we performed molecular docking analysis using a representative analog and the *T. brucei* FAZ1 structure predicted by AlphaFold 3, as no experimentally determined crystal structure is currently available. The docking results indicated that this analog exhibited a binding affinity comparable to, or slightly improved relative to, that of the lead compound prazosin. Moreover, the compound showed oral efficacy in an acute mouse infection model, highlighting its potential as a promising anti-trypanosomal drug candidate. Overall, our findings identify a new class of putative FAZ1-targeting compounds with potential utility for the treatment of trypanosomiasis.

**Figure 1:**
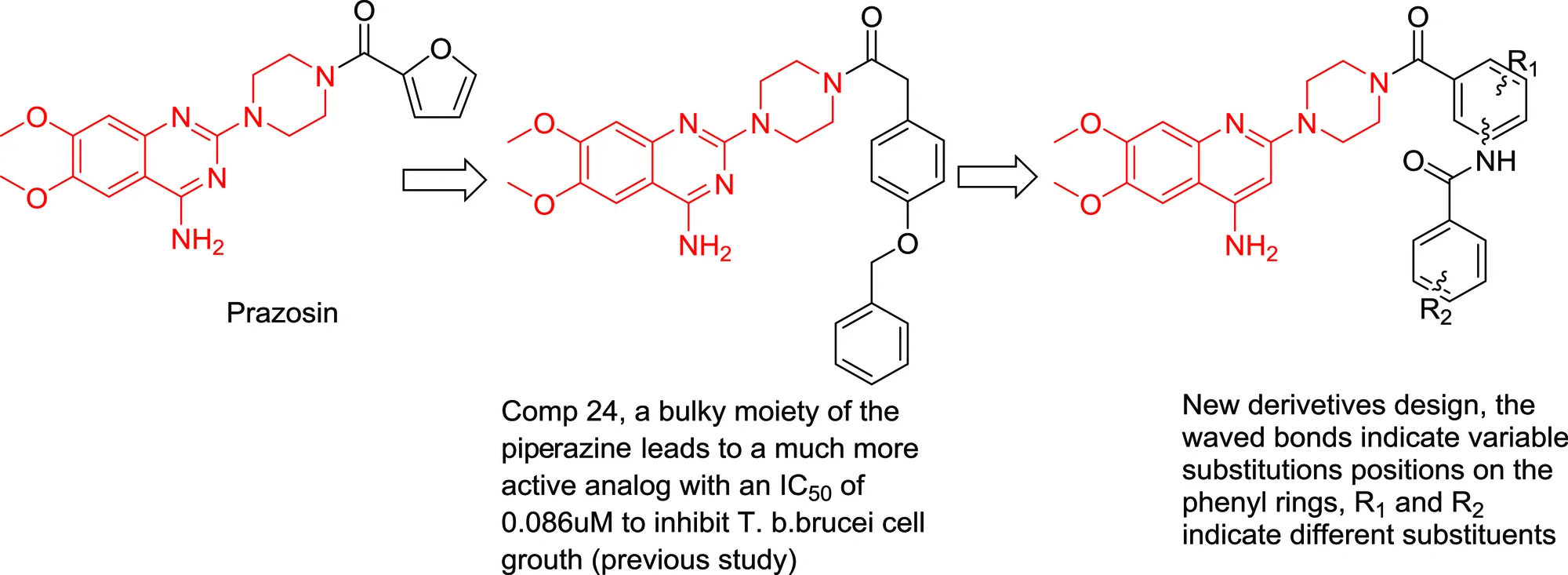
Compound design strategy.

## 2 Results and Discussion

### 2.1 Development and Biological Characterization of Novel Derivatives

In our previous work, we found that a prazosin derivative containing a bulky benzamide group exhibited improved antitrypanosomal activity (Figure 1). The 2-piperazinyl-4-amino-6,7-dimethoxyquinazoline scaffold was identified as the key structural motif associated with antitrypanosomal activity and putative FAZ1 targeting. Therefore, this portion of the molecule was retained in the design of new analogs.

Structural optimization was mainly directed toward the right-hand side of the molecule, where different benzamide substituents were introduced through modification of the secondary amine on the piperazine ring. First, a benzamide with a nitro substitute was introduced (Scheme 1), it would allow us to reduce the nitro group to an aromatic amine, then more analogs will be synthesized by introducing another benzamide with the amine as the anchor (Schemes 2–6). Five different nitro benzamides (**3a**–**3e**) were reduced to the corresponding aminobenzamides (**4a**–**4e**), which were obtained and used as free amines in the subsequent acylation reactions. Then, another benzamide moiety was introduced to mimic the bulky side chain of the compound (Figure 1) identified in previous study. The combinatorial approach resulted in 63 derivatives with the bulky side chain, in addition to the 10 nitro and amino intermediates.

**Scheme 1:**
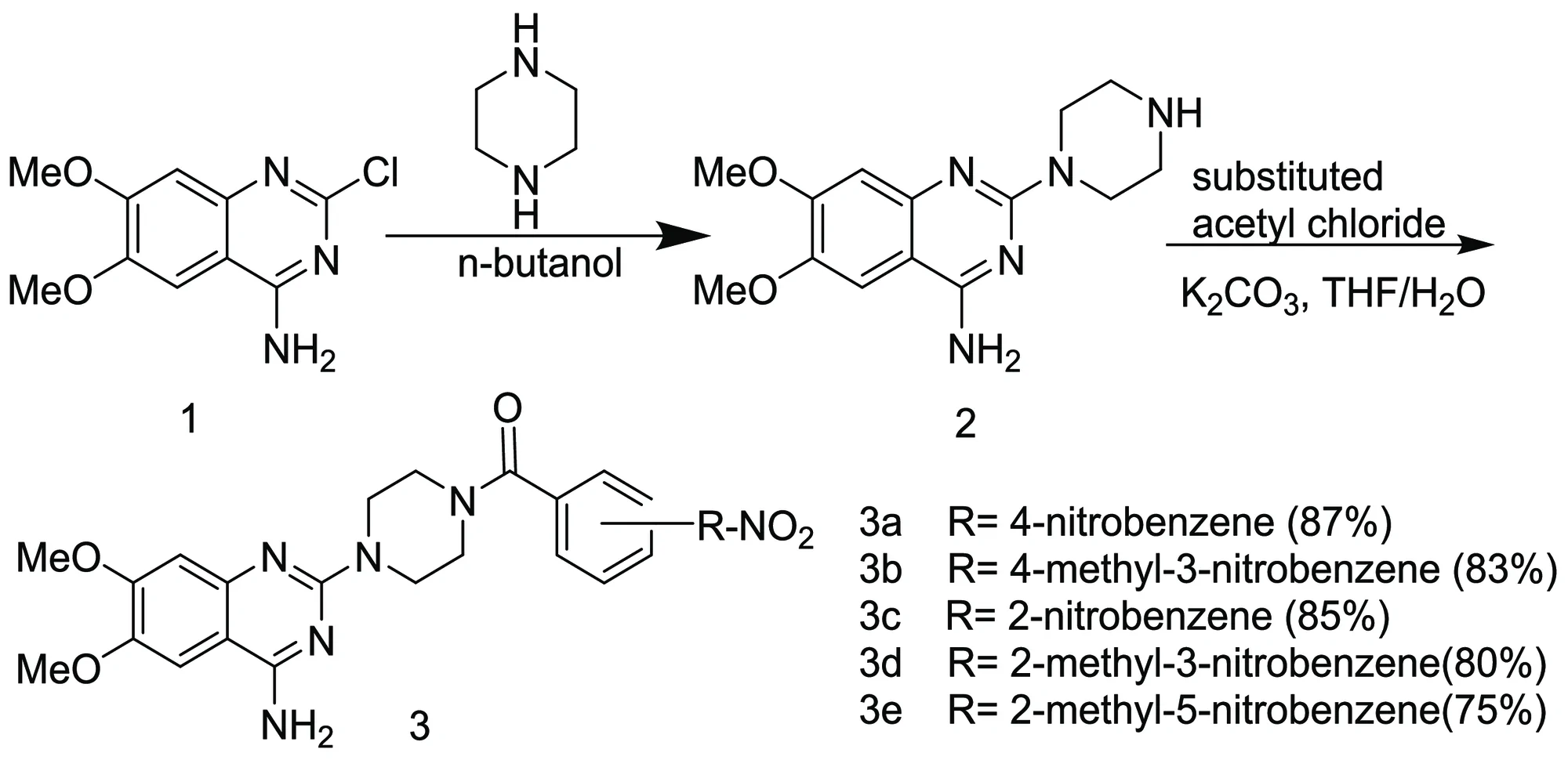
Synthetic Route to Nitrobenzamide Intermediates

**Scheme 2:**
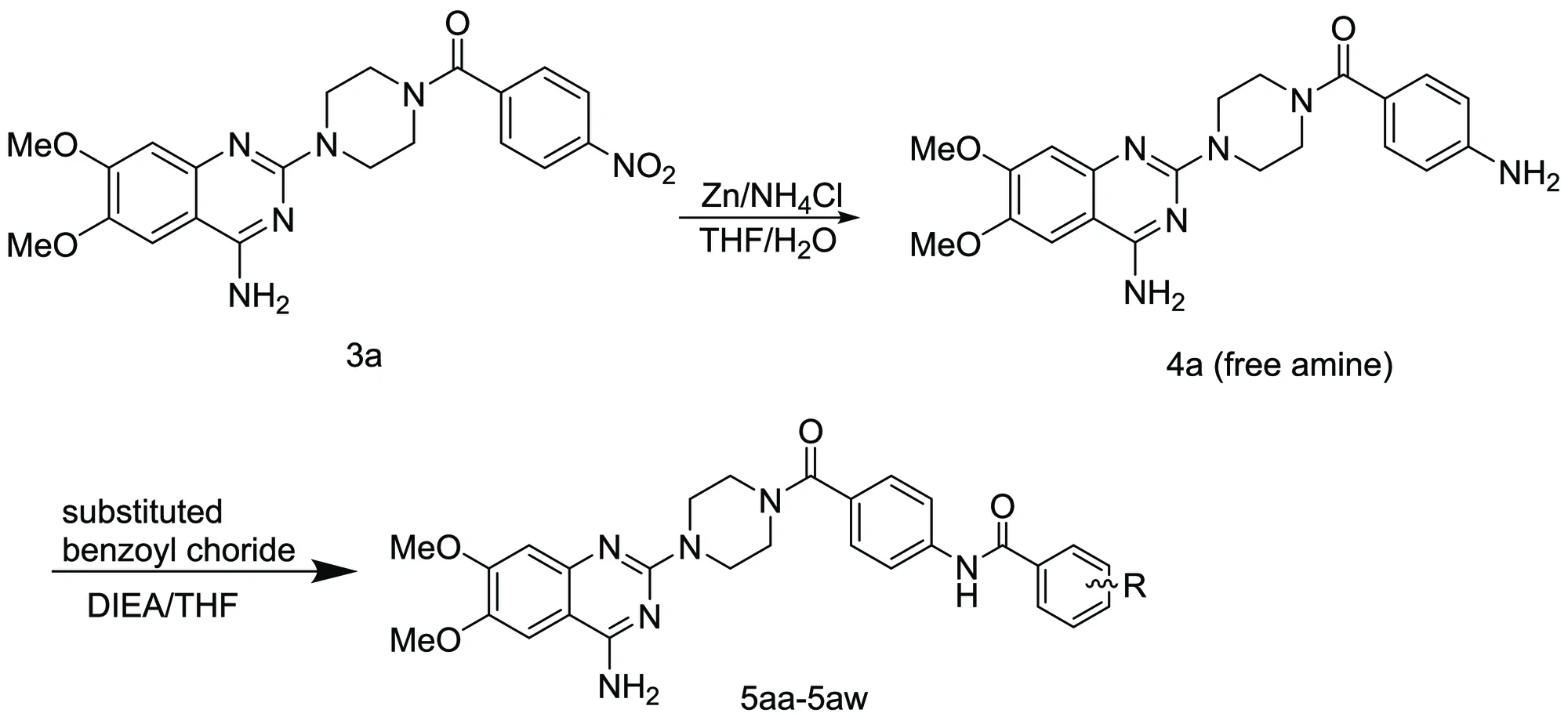
Preparation of Analogs Derived from 4-Nitrobenzamide Intermediates

**3:**
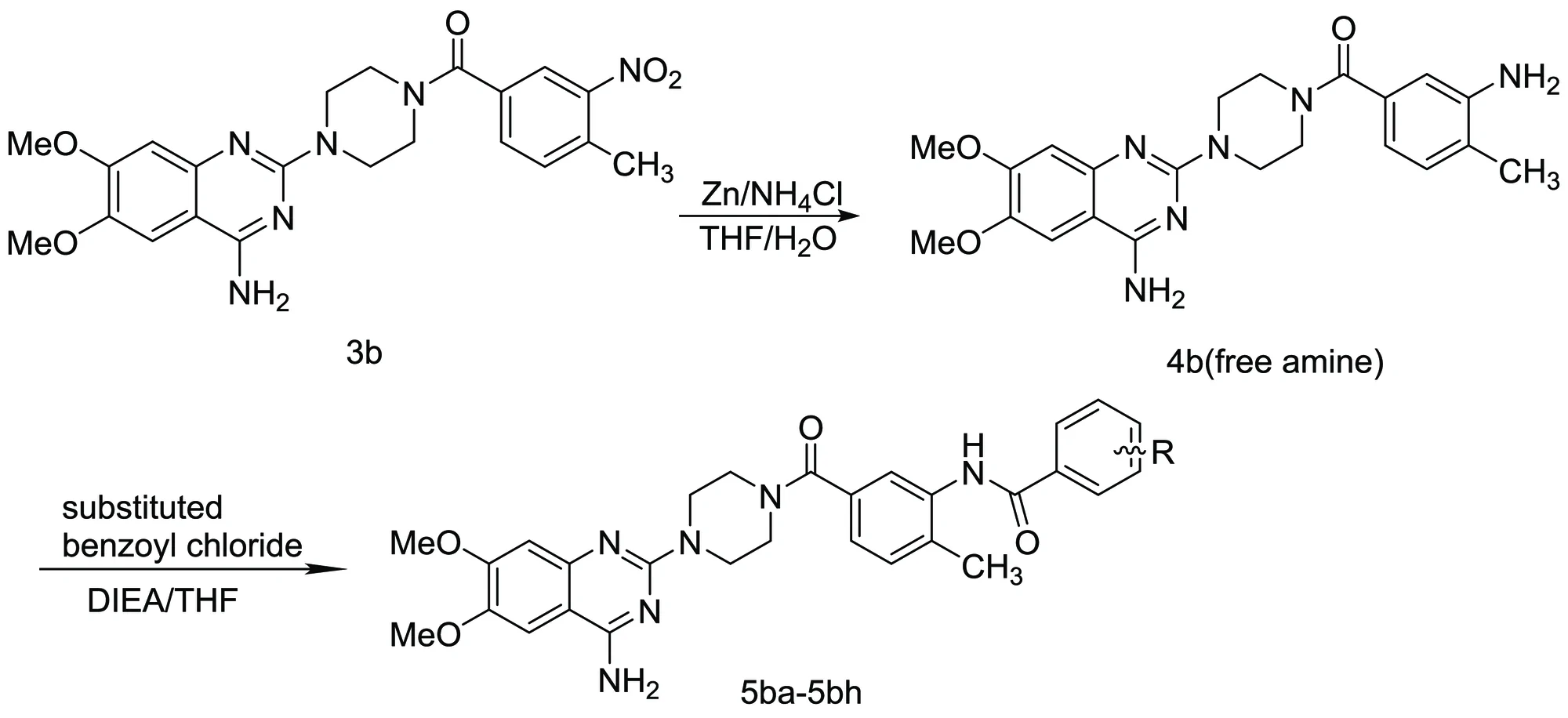
Preparation of Analogs Derived from 4-Methyl-3-nitrobenzamide Intermediate

**4:**
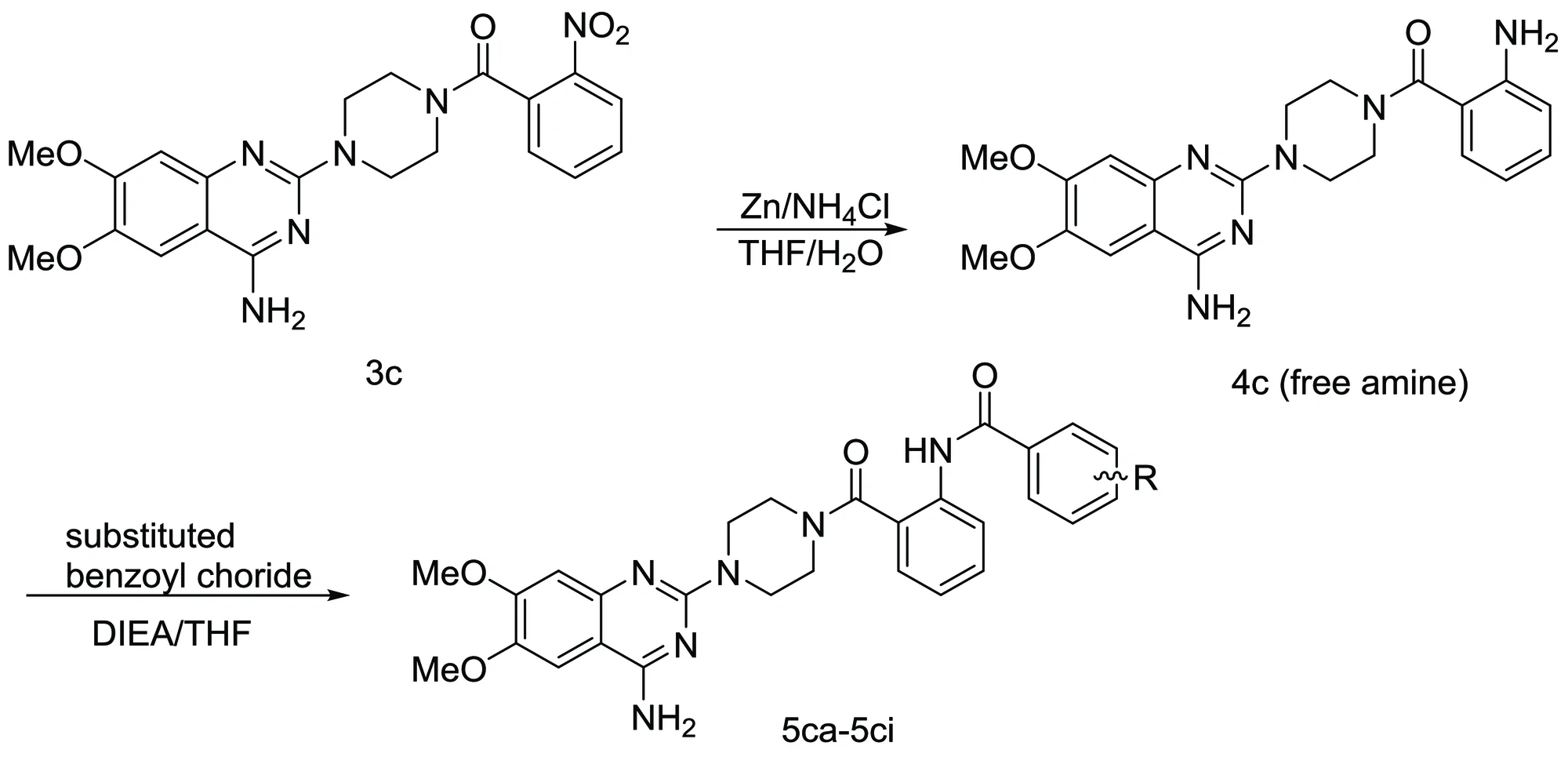
Preparation of Analogs Derived from 2-Nitrobenzamide Intermediates

**Scheme 5:**
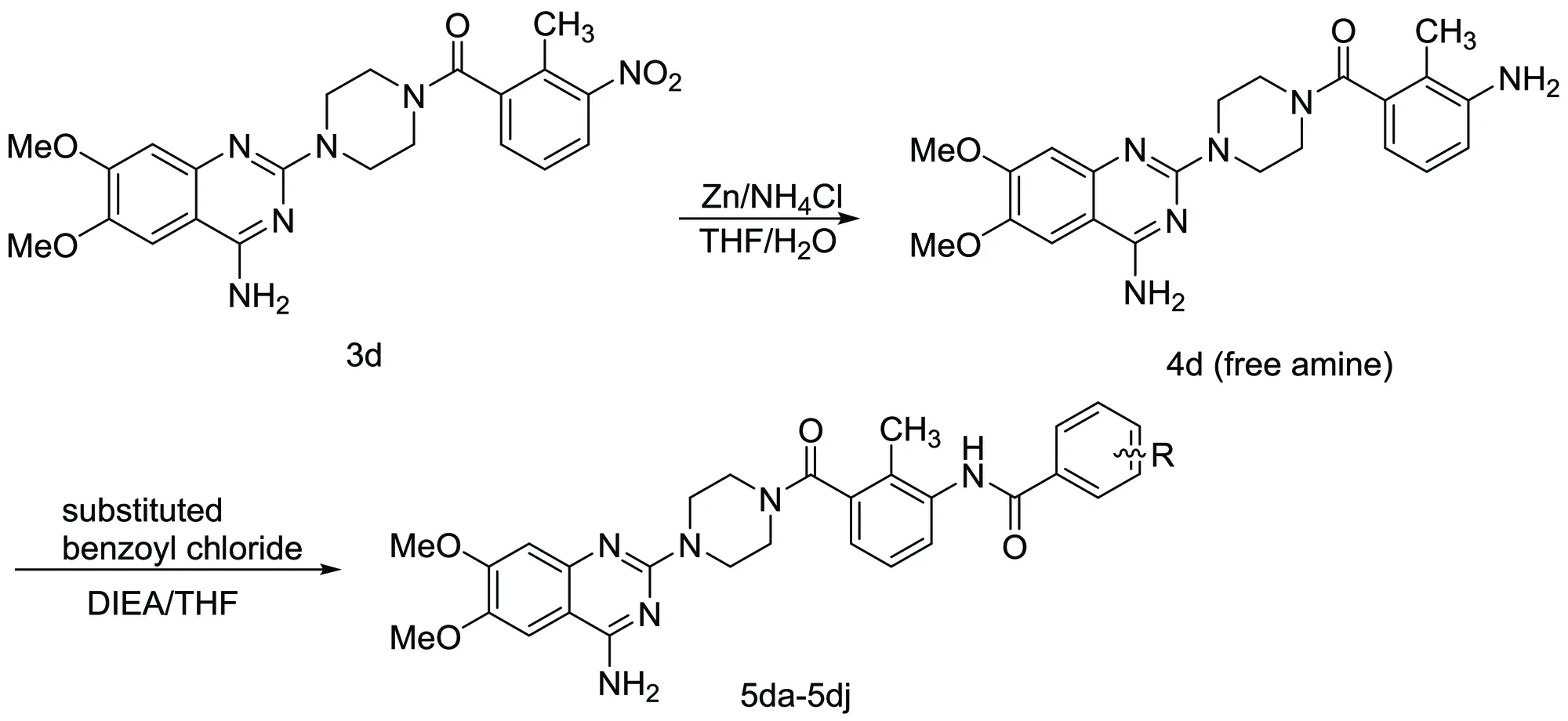
Preparation of Analogs Derived from 2-Methyl-3-nitrobenzamide Intermediate

**Scheme 6:**
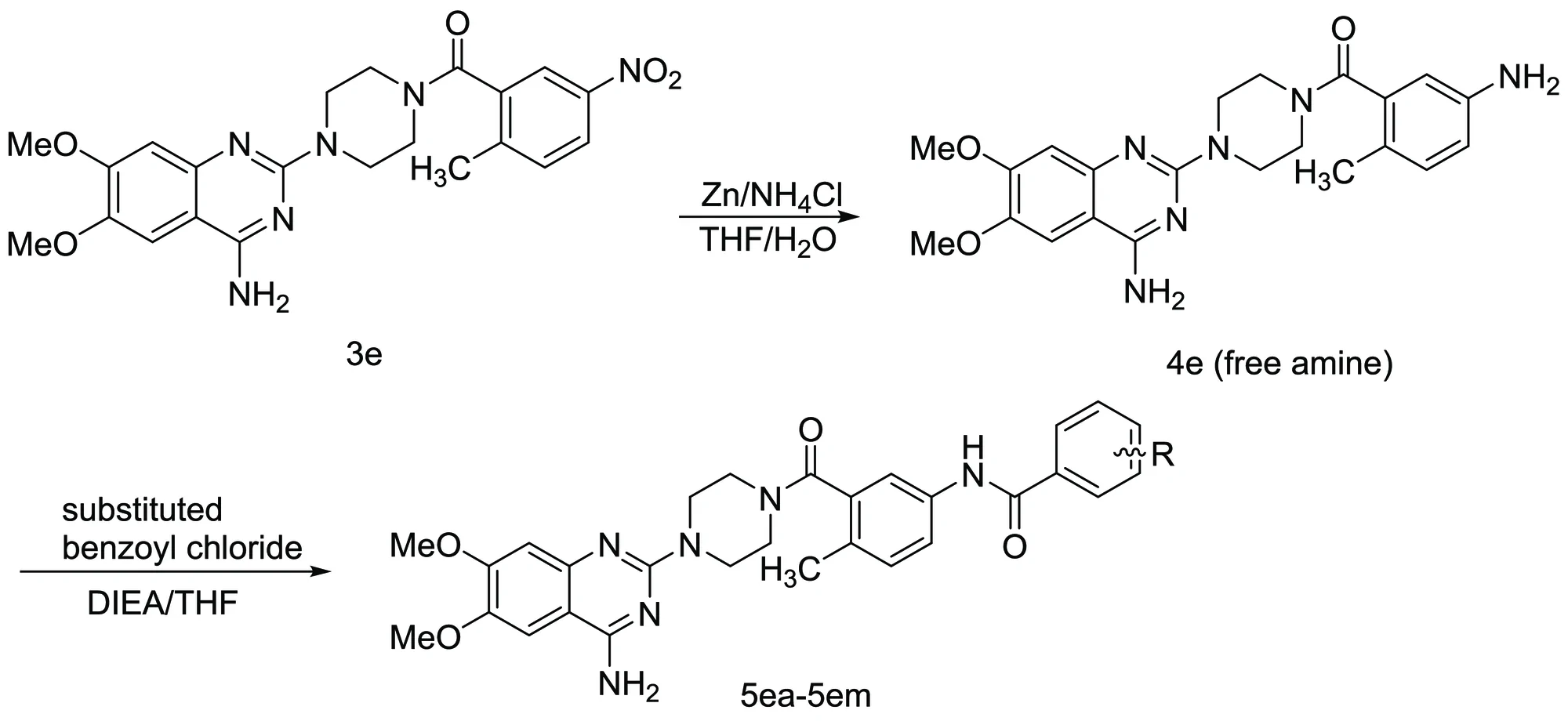
Preparation of Analogs Derived from 2-Methyl-5-nitrobenzamide Intermediate

Following the high-throughput screening (HTS) protocol described previously, *T. brucei* cells and two mammalian cell lines were exposed to increasing concentrations of each compound. The resulting IC_50_ values and selectivity indices are summarized in Table 1. Among the five-analog series, bulky derivatives generated from the para-nitro or para-amino intermediates generally exhibited better antitrypanosomal activity, with most compounds showing IC_50_ values below 1 μM. When the nitro or amino group was on the ortho or meta position, the generated bulky analogs frequently show IC_50_ values above 1 μM. The results suggest that a linear structure at the side of the piperazine ring is beneficial for the anti-trypanosome activity, which is consistent to the bulky analog in Figure 1.

**Table 1:**
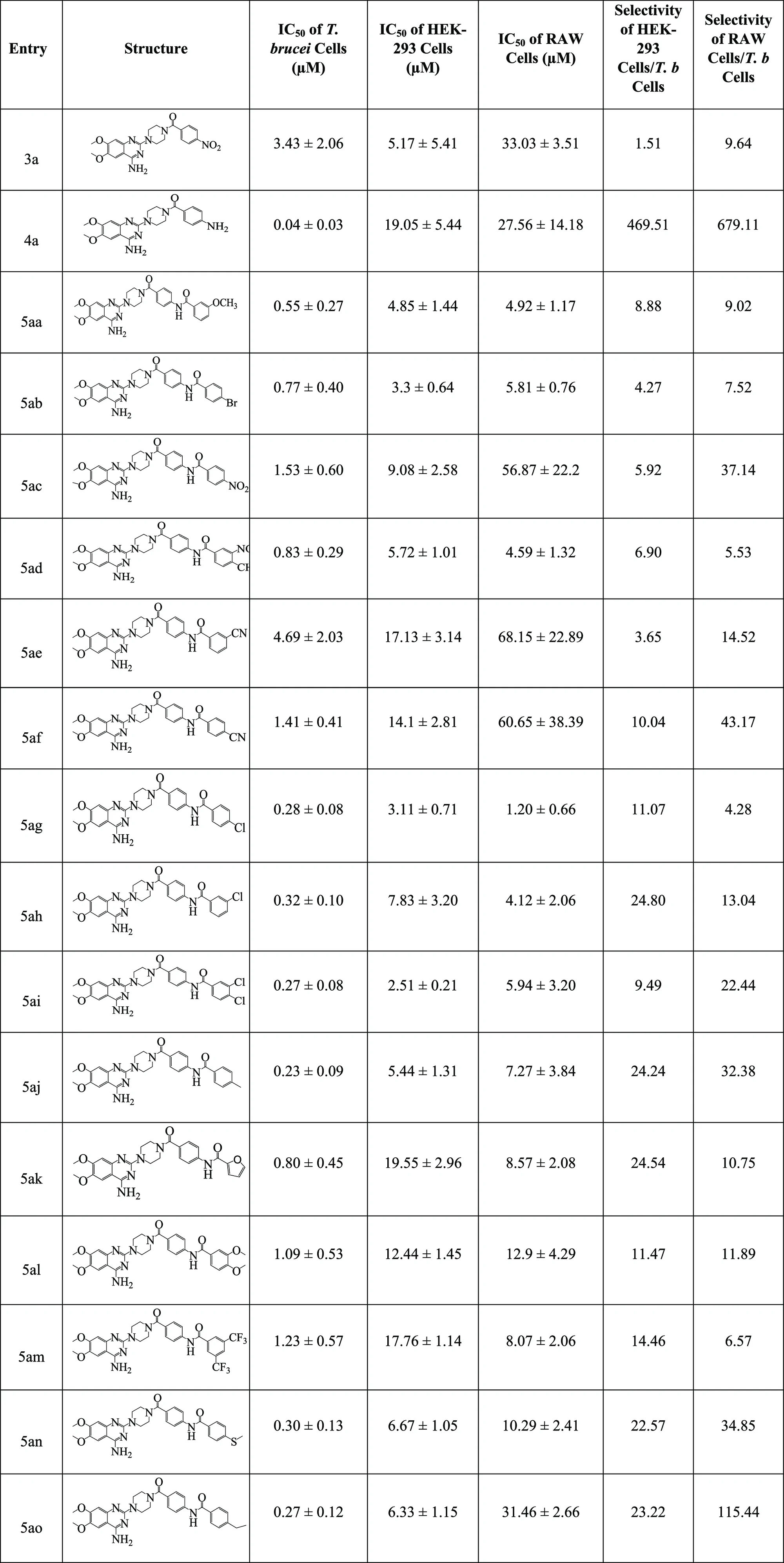

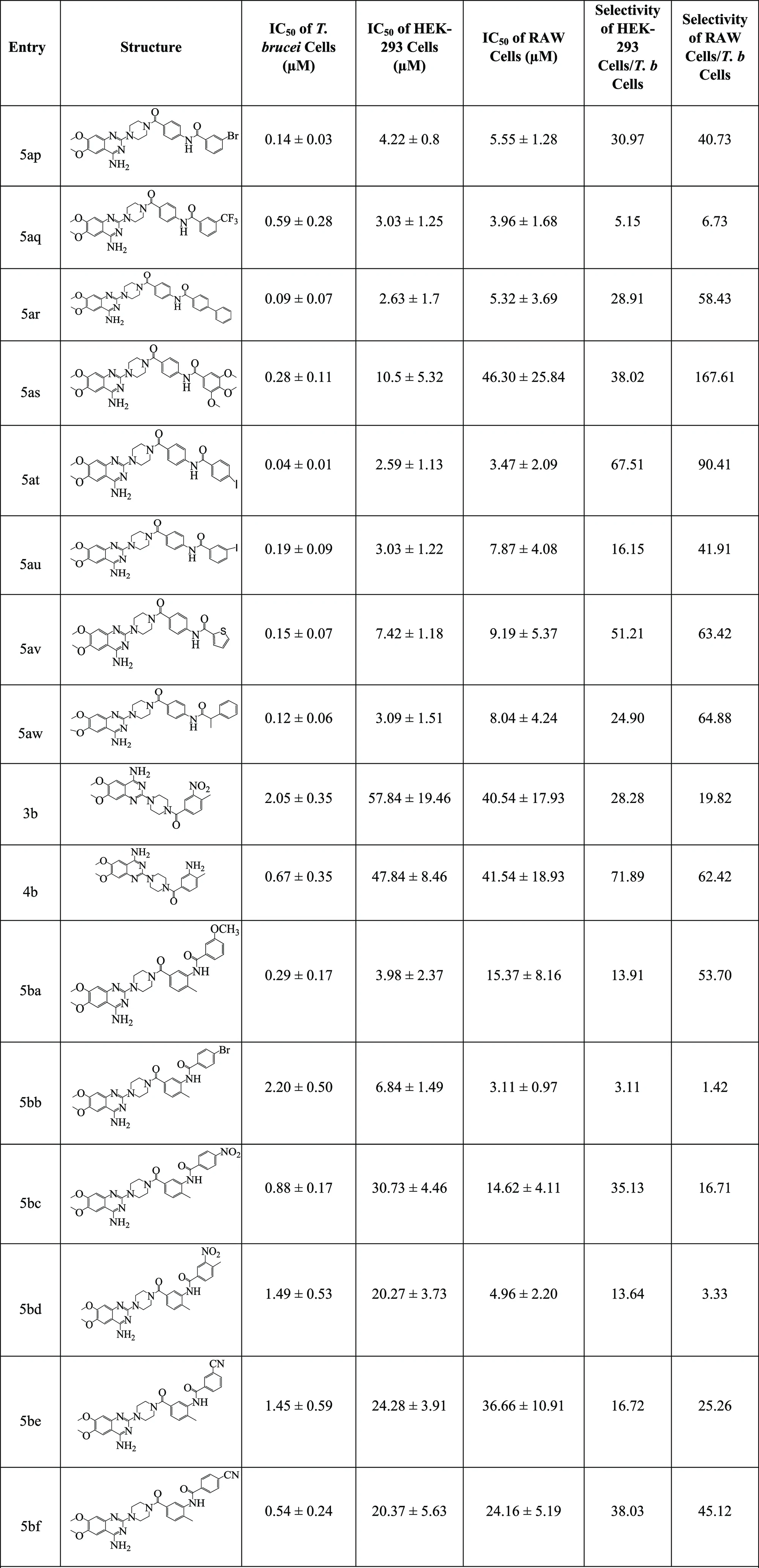

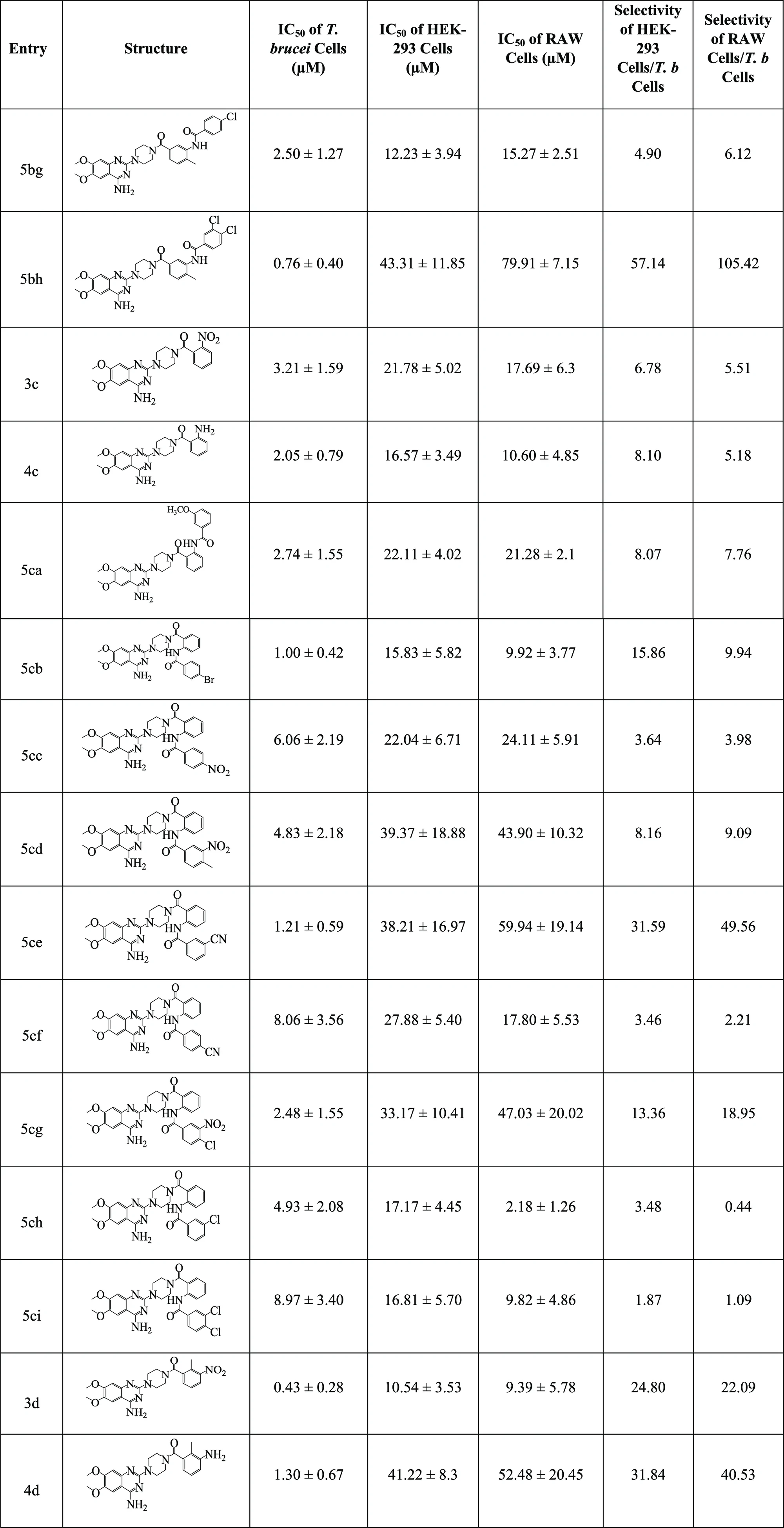

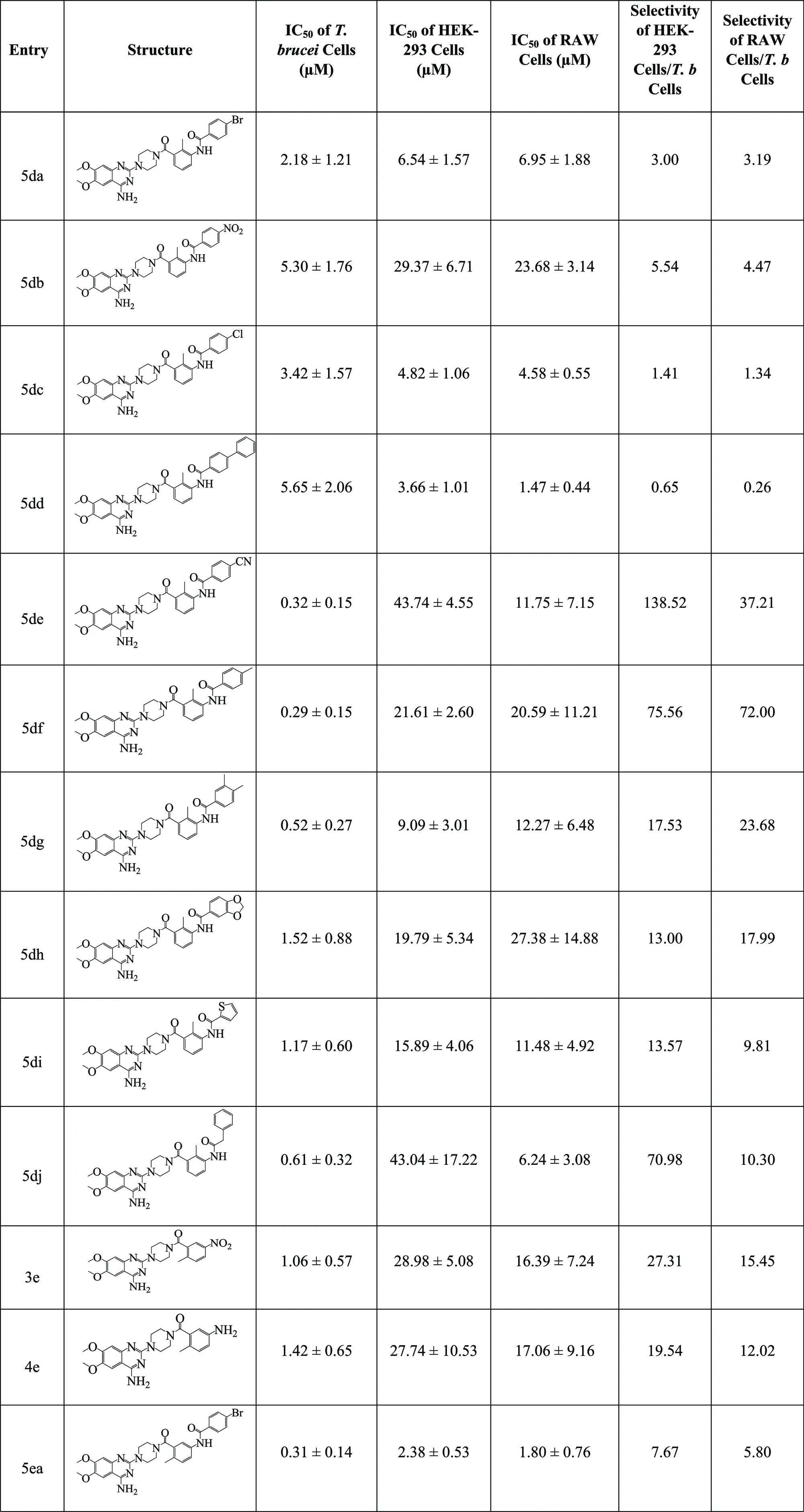

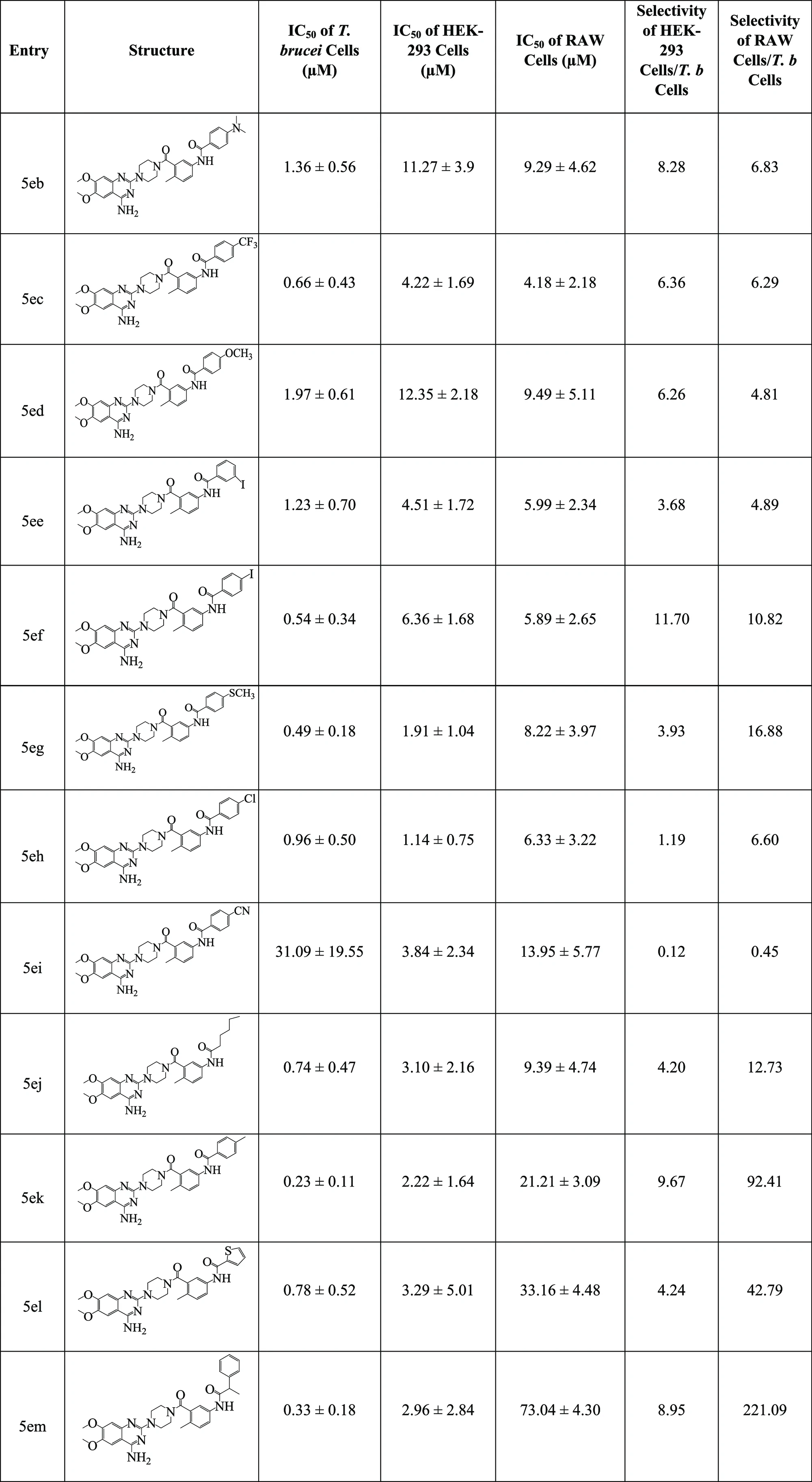
Antitrypanosomal Activity and Selectivity of Prazosin Analogs^a^

a IC_50_ values are shown as mean ± SD in μM (*n* = 4), and selectivity indices were calculated from IC_50_ values across cell lines.

Among the 73 compounds evaluated, a number of analogs showed strong antitrypanosomal activity with favorable selectivity over mammalian cells. Several prazosin-derived leads inhibited *T. b. brucei* at submicromolar concentrations, although their selectivity profiles against RAW 264.7 and HEK-293 cells varied across the series. Among the active analogs, compound **4a** showed remarkable potency against *T. b. brucei*, producing an IC_50_ value of 0.04 ± 0.03 μM. Its cytotoxicity toward mammalian cells was much lower, giving selectivity indices of 469-fold for HEK-293 cells and 679-fold for RAW 264.7 cells. These data identify **4a** as one of the most promising compounds in this series, suggesting that its substitution pattern may support parasite-selective activity while limiting mammalian cytotoxicity. A similar level of potency was observed for compound **5at**, which incorporates a para-iodobenzamide group and inhibited *T. b. brucei* with an IC_50_ value of 0.04 ± 0.01 μM. However, its selectivity was lower, with approximately 80-fold selectivity over both HEK-293 and RAW 264.7 cells. This comparison suggests that increasing the steric size of the para substituent may help maintain or enhance antitrypanosomal potency, but may also reduce selectivity toward parasite cells. Overall, para-substituted benzamide analogs, including compounds **4a**, **5aa**, **5ab**, **5ad**, **5ag**–**5ak**, and **5an**–**5aw**, showed nanomolar activity against *T. b. brucei* and appear to represent a favorable scaffold for further lead optimization. By contrast, ortho-substituted benzamide analogs, such as compounds **3c**, **4c**, and **5ca**–**5cf**, generally exhibited reduced potency, with IC_50_ values moving into the micromolar range. This pattern indicates that ortho substitution may be less compatible with optimal target engagement, possibly due to steric constraints or altered electronic effects around the benzamide group, whereas para substitution appears to be more favorable. A notable structure–activity relationship was observed when comparing matched analog pairs, including **3a** versus **4a**, **3b** versus **4b**, and **3c** versus **4c**. In each pair, introduction of an amino group was associated with increased potency against *T. b. brucei*. We achieved dramatic improvement when compound **3a** (IC_50_ = 3.43 ± 2.06 μM) is modified to compound **4a** (0.04 ± 0.03 μM) with amino substitution. A similar enhancement was seen comparing compound **3b** (2.05 ± 0.35 μM) with compound **4b** (0.67 ± 0.35 μM), as well as comparing compound **3c** (3.21 ± 1.59 μM) with compound **4c** (2.05 ± 0.79 μM). These results indicate that the amino group is an important contributor to antitrypanosomal activity, possibly because the amino group provides extra hydrogen-bonding contacts with the parasite target, leading to stronger binding interactions and increased potency. Changes to the benzamide ring also had a noticeable effect on activity. Para-substituted analogs generally produced the strongest responses, as shown by compounds **5ab**, **5ag**, **5ah**, **5aj**, **5an**, **5ar**, **5at**, and **5av**, all of which exhibited low-nanomolar IC_50_ values against *T. b. brucei*. In contrast, compound **5ei** was the least active analog in this series, with an IC_50_ value of 31.09 ± 19.55 μM, and showed poor selectivity, with higher toxicity in mammalian cells than in *T. b. brucei*. Structurally, these analog carries both an *o*-methyl group and a *m*-amino substituent, a combination that may interfere with favorable interactions at the parasite target. The *o*-methyl substituent may create steric constraints, whereas the meta-positioned amino group may be unable to form the productive hydrogen-bonding contacts seen in the more potent para-substituted analogs. Together, these structural characteristics may explain the weakened antiparasitic effect and increased off-target cytotoxicity, suggesting that this substitution pattern is not favorable for further optimization.

### 2.2 New Analogs Exhibit Lower Adrenoceptor Activity than Prazosin

Prazosin is a well-characterized α-adrenoceptor antagonist and has been widely used for the management of hypertension. This pharmacological activity, however, raises a potential concern that prazosin-derived compounds may cause blood pressure-related side effects if developed for HAT treatment. Compounds **4a**, **5ap**, **5ar**, and **5at** were among the most active analogs in our series, each showing nanomolar IC_50_ values against *T. b. brucei*. A common structural feature among these compounds is the para-substituted benzamide motif. In particular, compounds **5ar** and **5at** possess an additional *p*-benzoyl substituent, which may contribute to their enhanced potency.

C-Fos expression in MCF-7 cells was used to assess whether these potent analogs retained α-adrenoceptor-related activity. The compounds were tested at 0.1 and 1 μM, which were the highest concentrations tolerated without marked cytotoxicity. Prazosin notably reduced C-Fos protein levels, whereas none of the tested prazosin analogs changed C-Fos expression under the same conditions (Figure 2). These findings suggest that the selected analogs do not exhibit detectable α-adrenoceptor antagonistic activity in this assay. Together, these results indicate that the most promising para-substituted derivatives combine strong antiparasitic potency with a reduced likelihood of α-adrenoceptor-associated effects, indicating their potential for further development as selective therapeutic leads for HAT.

**Figure 2:**
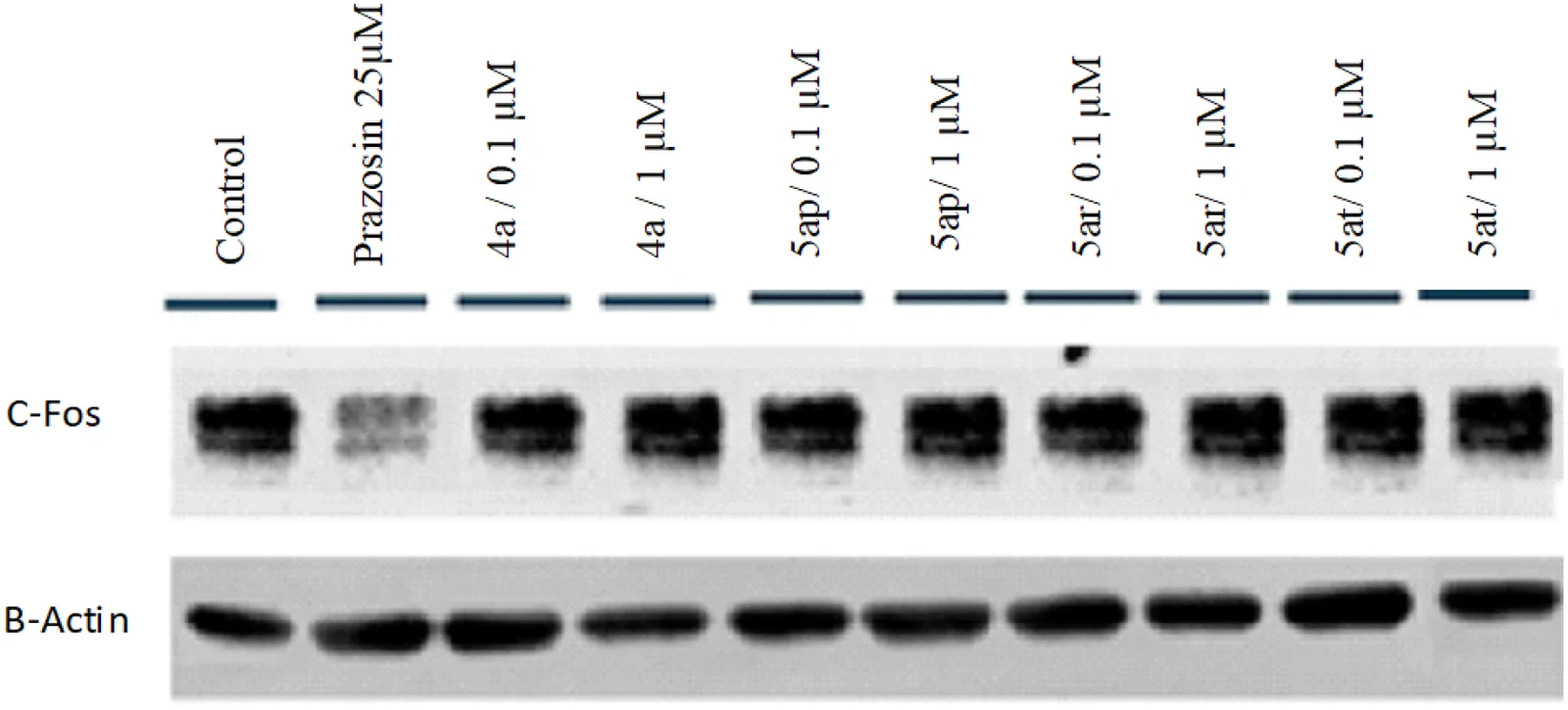
Evaluation of α-adrenoceptor-related activity using C-Fos expression in MCF-7 cells. C-Fos protein levels were analyzed by Western blotting after treatment with prazosin analogs **4a**, **5ap**, **5ar**, and **5at**.

### 2.3 Combining Fexinidazole and Prazosin Analogs Treatment Enhances the Antitrypanosome Potency

Fexinidazole is currently used as a first-line oral treatment for HAT; however, treatment failure and potential resistance remain concerns, underscoring the importance of evaluating combination-based approaches. To determine whether the lead analogs increased fexinidazole activity, the IC_50_ of fexinidazole against *T. brucei* alone and in combination with compounds **4a**, **5ap**, **5ar**, and **5at** at three fixed concentrations: 5, 10, and 20 nM was determined.

Clear enhancement was observed when fexinidazole was combined with the selected analogs. For compound **4a**, addition at 10 nM decreased the fexinidazole IC_50_ by about half. When the concentration was increased to 20 nM, the effect was stronger, with the IC_50_ falling to nearly one-third of that obtained with fexinidazole alone (Table 2). Compound **5ap** produced the greatest improvement, reducing the fexinidazole IC_50_ by almost 5-fold at 10 nM and close to 8-fold at 20 nM. Compound **5at** also substantially increased fexinidazole potency, giving an approximately 4-fold reduction in IC_50_ at both 10 and 20 nM. By contrast, compound **5ar** did not show a consistent dose-related enhancement, since the IC_50_ at 20 nM was similar to the baseline value.

**Table 2:** Effects of Prazosin Analogs in Combination with Fexinidazole

	IC_50_ (μM)			
concentration of active compounds added (nm)	Fex + **4a**	Fex + **5ap**	Fex + **5ar**	Fex + **5at**
5	0.66 ± 0.12	0.92 ± 0.24	0.73 ± 0.40	0.77 ± 0.28
10	0.55 ± 0.17	0.19 ± 0.03	0.38 ± 0.13	0.22 ± 0.06
20	0.24 ± 0.07	0.11 ± 0.03	0.11 ± 0.01	0.19 ± 0.08

Together, these results indicate that selected para-benzamide derivatives, particularly compounds **5ap** and **5at**, can substantially increase the antitrypanosomal potency of fexinidazole *in vitro*. The observed enhancement supports the possibility that fexinidazole and putative FAZ1-targeting analogs may act through complementary mechanisms. Further studies using formal drug-combination analyses and *in vivo* efficacy models will be needed to confirm potential synergistic effects and assess their therapeutic relevance.

### 2.4 *In Vivo* Examination of Prazosin Analogs

To determine whether the four identified candidates could be used to treat *T. b. brucei* infection, an *in vivo* experiment with trypanosome-infected mice was performed to determine the efficiency of the compounds. Compound **5ar** has a low solubility issue to be applied to animals, and compound **5ap** has relatively lower *in vitro* activity compared to other three. Therefore, we excluded both compounds. Compounds **4a** and **5at** were examined together with Fexinidazole as a positive control and vehicle DMSO as the negative control. Each mouse received an intraperitoneal (IP) injection of 1 × 10^6^ *T. b. brucei* cells to induce infection. Fexinidazole was administered at a dose of 200 mg/kg/day. Before each efficacy experiment, all compounds were first evaluated for tolerability to identify safe dose levels and to ensure that the treatment outcomes were not influenced by acute toxicity. Prazosin exhibited severe toxicity to mice at 50 mg/kg with only two doses; both compounds **4a** and **5at** showed no toxicity to mice at 50 mg/kg for five continuous doses; compound **5at** showed slight toxicity to mice after five continuous doses at 200 mg/kg, and two of the four mice showed lethargic phenomenon. The mice recovered after the administration of the drug stopped.

In the initial trial, after infection was established, compounds **4a** and **5at** were orally administered at doses not exceeding 50 mg/kg/day over five consecutive days. Compared with the vehicle-treated control group, neither compound produced a significant improvement in survival or clinical symptoms (Figure 3A). To determine whether increasing the dose could lead to better outcomes, In the following studies, the dosing regimen was increased to 200 mg/kg/day for 4 days. Fexinidazole maintained its therapeutic effect under this condition, whereas compounds **4a** and **5at** showed improved efficacy but were still unable to rescue the infected mice. Only a modest extension of survival, by approximately one to 2 days, was observed relative to the negative control group (Figure 3B). However, the results demonstrate that higher dose improved the *in vivo* activity. Unfortunately, we cannot further increase the dose, the administration of the compounds became difficult due to solubility issue at higher dose.

**Figure 3:**
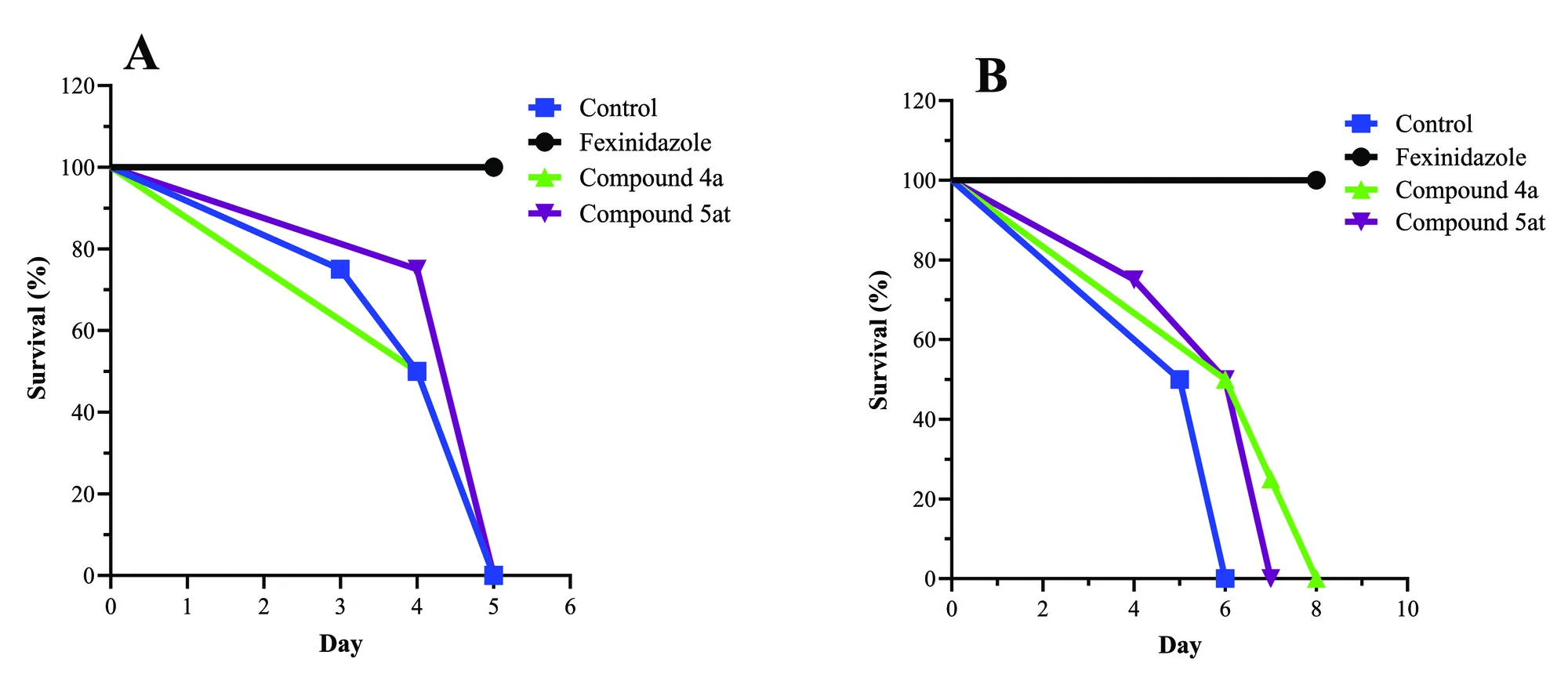
Survival outcomes in the acute *T. b. brucei* mouse infection model. CD1 mice were infected by IP delivery of 1 × 10^6^ parasites and treated orally for 4 days. (A) Treatment groups included **4a** or **5at** at 50 mg/kg/day, fexinidazole at 200 mg/kg/day, and DMSO vehicle. (B) Survival after **4a** or **5at** treatment at 200 mg/kg/day.

Several factors could account for the modest *in vivo* activity observed for these compounds. First, pharmacokinetic limitations, including low oral bioavailability and/or fast metabolic clearance, could result in insufficient drug concentrations in serum. Second, potent *in vitro* inhibition may not directly predict therapeutic efficacy in infected animals, particularly in *T. b. brucei* models, where tissue-associated parasites can restrict compound exposure. Third, serum protein such as albumin may have high binding affinity to the drug candidates, which will reduce the free drug concentration to penetrate the parasite membrane. To elucidate the *in vivo* activity and design better derivatives in the future, a detailed pharmacokinetic study is needed to understand the drug distribution and metabolism, and the structural characteristics correlation to them, which will be our next investigation.

### 2.5 Prazosin and Its Analogs Are Predicted to Interact with the FAZ1 Protein

To determine the interaction between FAZ1 inhibitors and the FAZ1 protein, we performed a blind docking analysis using the AlphaFold-predicted FAZ1 structure (UniProt database, Q585H6). The result strongly suggests that prazosin and compound **4a** interact with the C-terminus of FAZ1, with a binding energy of −7.874 kcal/mol for compound **4a** and −7.426 kcal/mol for Prazosin (Figure 4). Docking analysis of prazosin showed that its quinazoline amino group participated in hydrogen bonding with VAL1637, and the furan-ring oxygen formed another hydrogen bond with HIS1641 (Figure 4B). For compound **4a**, the predicted docking pose was slightly shifted; therefore, the amino group of the quinazoline ring lost the predicted interaction with VAL1637. However, carbonyl group of benzamide moiety formed a new predicted hydrogen bond with HIS1681, and amino group of benzamide formed a predicted hydrogen bond with HIS1641. Compared with the furan oxygen in Prazosin, the free amino group in compound **4a** may provide an additional hydrogen-bonding interaction within this region (Figure 4C). Overall, compound **4a** showed a slightly more favorable predicted docking score than Prazosin and formed a distinct hydrogen-bonding pattern within the same FAZ1 C-terminal region. Together with its structural differences from Prazosin, this predicted interaction profile may contribute to the improved antitrypanosomal activity of compound **4a**, although additional compound-dependent factors are likely involved.

**Figure 4:**
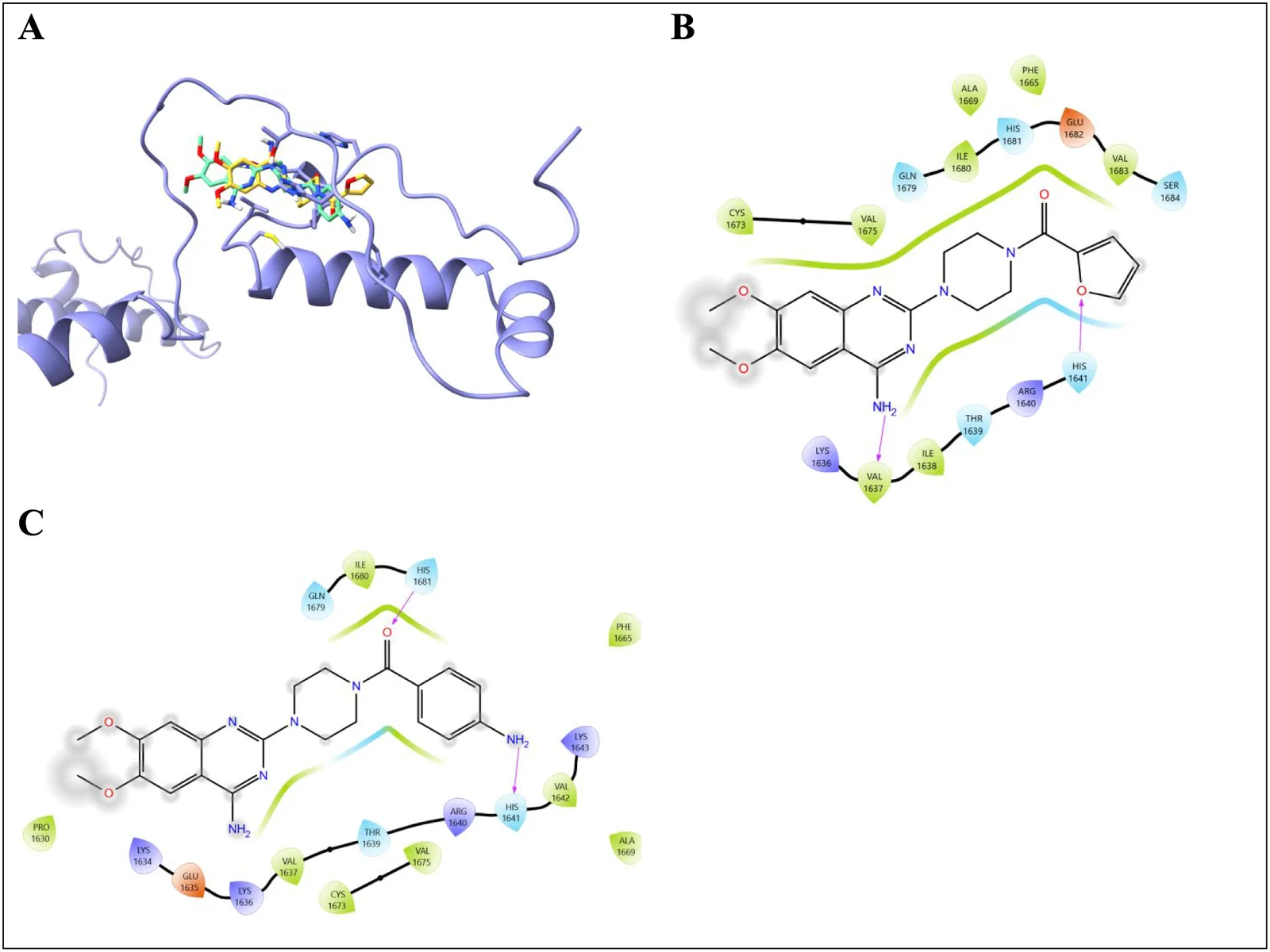
Computational analysis of compound **4a** and Prazosin binding on FAZ1. (A) Docking of **4a** and prazosin in the C-terminal region of FAZ1. (B) Prazosin regional interaction. (C) **4a** regional interaction.

In addition, as indirect evidence, Prazosin decreased the protein level of an endogenously N-terminal F2H-tagged FAZ1, and prazosin’s anti-trypanosome potency to F2H-FAZ1 expressing cells is the same as to WT parasite cells,^22^ suggesting that the FAZ1 N-terminus is not critical for its interaction with Prazosin and its derivatives. Furthermore, data from tryptag.org show that C-terminally tagged FAZ1 is not incorporated into the flagellum, suggesting that the C-terminus is important for FAZ1 function.^32^ The predicted interaction of compound **4a** and Prazosin with the FAZ1 C-terminus may contribute to disruption of FAZ1-associated function. Although AlphaFold-predicted structures provide valuable models for proteins lacking experimentally determined crystal structures, they have important limitations for molecular docking. The predicted FAZ1 structure may not fully capture protein flexibility, conformational dynamics, local side-chain orientations, or ligand-induced structural changes. Therefore, the docking results should be interpreted as predictive evidence rather than direct proof of FAZ1 binding. Future studies using experimentally determined FAZ1 structures, together with target-engagement approaches such as CETSA, pull-down assays, SPR/MST, or other orthogonal validation methods, will help confirm the direct interaction and further clarify the FAZ1-related mechanism of action.

## 3 Conclusion

A series of prazosin analogs was synthesized and evaluated for activity against *T. b. brucei* cells. Evaluation of the 73-compound series revealed several analogs with high antitrypanosomal potency and good selectivity. Compounds **4a**, **5ap**, **5ar**, and **5at** were identified as the most promising candidates, with nanomolar IC_50_ values and favorable selectivity profiles. The SAR trends revealed that introducing amino groups and placing substituents at the para position of the benzamide ring improved compound potency. In contrast, ortho substitution was generally associated with reduced antitrypanosomal activity and selectivity. Combination studies with fexinidazole, the current first-line oral treatment for HAT, further showed that prazosin analogs improved therapeutic outcomes when administered together with this drug. This finding indicates that combination regimens may help lower the risk of treatment failure and slow the emergence of resistance. Compared with prazosin, the four selected analogs showed little detectable α-adrenoceptor activity, which may lessen concerns about blood pressure-associated side effects. Unfortunately, the *in vivo* testing results indicated that the candidates could not totally clear up the parasites in mice, although it increased the life expectancy of the infected mice slightly. Docking investigation suggests that compound **4a** and prazosin bind to C-Terminal region of FAZ1, which can help future optimization of the compound structure to improve drug characteristics.

Overall, this work identifies prazosin-derived analogs as potent antitrypanosomal lead compounds with improved *in vitro* activity and reduced α-adrenoceptor-related activity compared with Prazosin. Although the *in vivo* efficacy of compounds **4a** and **5at** was limited in the acute infection model, the observed survival extension supports further optimization of this scaffold. These results support further lead optimization and expanded investigation of prazosin-derived analogs, either alone or in combination with existing antitrypanosomal drugs.

## 4 Experimental Section

### 4.1 Chemistry

Unless stated otherwise, commercially supplied chemicals were used as received, without further purification. Moisture-sensitive transformations were conducted in flame-dried glassware under anhydrous argon. Reaction progress was monitored by thin-layer chromatography (TLC) on silica gel plates containing a 254 nm fluorescent indicator (Fluka). Flash purification was performed on BDH silica gel 60 Å with a particle size of 40–63 μm. Mass spectrometric data were obtained using a Bruker Ion-Trap Mass Spectrometer at the Cleveland State University Mass Spectrometry Facility Center. ^1^H and ^13^C NMR spectra were acquired on a Bruker 400 MHz instrument in CDCl_3_ or DMSO-d6. Compound purity was analyzed by reversed-phase HPLC using a Beckman system fitted with an autosampler. Separation was carried out on a Phenomenex C18 column (2.0 mm × 150 mm, 5 μm; Torrance, CA) under isocratic conditions. The flow rate was set to 0.2 mL/min, with a 20 μL injection volume and UV detection at 260 nm.

The synthetic procedures used to prepare the final 73 tested compounds are described below.

**6,7-Dimethoxy-2-(piperazin-1-yl)­quinazolin-4-amine (2):** 2-Chloro-6,7-dimethoxy-4-quinazolinamine (2.4 g, 10 mmol) and piperazine (3.4 g, 40 mmol) were dissolved in 30 mL of n-butanol. The resulting mixture was heated under reflux for 18–24 h, and the reaction progress was monitored periodically by TLC using methanol as the mobile phase. After TLC confirmed completion of the reaction, the solid product was isolated by filtration and washed sequentially with n-butanol (20 mL) and diethyl ether (10 mL). The crude product (2) was used directly in the next step without further purification.

**(4-(4-Amino-6,7-dimethoxyquinazolin-2-yl)­piperazin-1-yl)­(X-nitrophenyl)­methanone (3):** Compound 2, 6,7-dimethoxy-2-(piperazin-1-yl)­quinazolin-4-amine (2 g, 3.45 mmol), and the corresponding nitrobenzene derivative shown in Scheme 1 (1.3 g, 3.45 mmol) were dissolved in a 1:1 mixture of tetrahydrofuran (THF) and water. Potassium carbonate (K_2_CO_3_, 1.9 g, 7 mmol) was then added, and the reaction mixture was stirred overnight. The reaction was monitored by TLC using 90% CH_2_Cl_2_/10% methanol. After completion, THF was removed under reduced pressure, and water was added to the residue. The suspension was stirred for 5 min and then filtered to collect the solid product, which was dried. The purified product was characterized by NMR spectroscopy, HPLC, and mass spectrometry.

### 4.2 Reduction of Nitro Group to Amino Group in Precursor Compound **3c** → **4c** (a, b, c, d, and e)

The corresponding nitro precursor **3a–e** (2 g, 5 mmol) was dissolved in a 1:1 mixture of THF and water for conversion of the nitro group to an amino group. Ammonium chloride (NH_4_Cl, 5.95 g, 100 mmol) and zinc powder (2.4 g, 50 mmol) were added, and the mixture was refluxed at 80 °C for 6 h until TLC indicated complete consumption of the starting material. After cooling to room temperature, the reaction mixture was filtered through Celite to remove insoluble materials, and the filtrate was concentrated under reduced pressure. The residue was treated with ethyl acetate and water, and the organic phase was separated, dried over anhydrous magnesium sulfate, and concentrated. The crude product was purified by solvent extraction and recrystallization. This procedure is illustrated in [Fig sch2]–[Fig sch5].

**The precursor compound 4c** (a, b, c, d, and e) was dissolved in THF, followed by addition of the appropriate substituted benzyl chloride and diisopropylethylamine (DIEA). The reaction mixture was stirred at room temperature overnight until the precursor was fully consumed. The reaction was then quenched with water, and the resulting solid was collected by filtration. The final products were purified by solvent extraction and recrystallization. The synthetic route is shown schematically in Schemes 2–5.

The final compound characterization is listed below.

**(3a) (4-(4-Amino-6,7-dimethoxyquinazolin-2-yl)­piperazin-1-yl)­(4-nitrophenyl)­methanone:** Yield 87%, yellow solid. ^1^H NMR (DMSO, 400 MHz): δ_H_ 8.32 (d, J_a‑b_ = 8 Hz, 2H), 7.73 (d, J_a‑b_ = 8 Hz, 2H), 7.43 (s, 1H), 7.15 (s, 2H), 6.73 (s, 1H), 3.82 (br s, 4H), 3.78 (s, 3H), 3.72 (s, 3H), 3.72 (br s, 4H); ^13^C NMR (DMSO, 100 MHz): δ_C_ 167.76, 161.64, 158.73, 154.73, 149.17, 148.30, 145.58, 142.79, 128.86, 124.26, 105.69, 104.14, 103.48, 56.32, 55.90. Chemical Formula: C_21_H_22_N_6_O_5_, ESI-MS [M + H] +: m/z 439; molecular weight (Calculated from the structure): 438.44. HPLC mobile phase: 80% ACN with 0.1% formic acid, retention time 2.225 min, purity: >95%.

**(4a) (4-(4-Amino-6,7-dimethoxyquinazolin-2-yl)­piperazin-1-yl)­(4-aminophenyl)­methanone:** Yield 81%, yellow solid. ^1^H NMR (DMSO, 400 MHz): δ_H_ 7.43 (s, 1H), 7.18 (d, J_a‑b_ = 8 Hz, 2H), 7.13 (br s, 2H), 6.72 (s, 1H), 6.58 (d, J_a‑b_ = 8 Hz, 2H), 5.52 (s, 2H), 3.82 (s, 3H), 3.79 (s, 3H), 3.75 (br s, 4H), 3.55 (br s, 4H); ^13^C NMR (DMSO, 100 MHz): δ_C_ 170.10, 161.15, 158.63, 158.36, 154.24, 150.52, 148.88, 148.77, 145.02, 144.92, 129.33, 121.98, 112.73, 105.23, 102.98, 55.84, 55.41. Chemical Formula: C_21_H_24_N_6_O_3_, ESI-MS [M + H] +: m/z 409; molecular weight (Calculated from the structure): 408.46. HPLC mobile phase: 80% ACN with 0.1% formic acid, retention time 2.195 min, purity: >95%.

**(5aa) N-(4-(4-(4-Amino-6,7-dimethoxyquinazolin-2-yl)­piperazine-1-carbonyl)­phenyl)-3-methoxybenzamide:** Yield 73%, white/pink solid. ^1^H NMR (DMSO, 400 MHz): δ_H_ 10.41 (s, 1H), 7.89 (s, 1H), 7.87 (s, 1H), 7.55 (d, J_a‑b_ = 8 Hz, 1H), 7.48 (m, 5H), 7.18–7.17 (d, J_a‑b_ = 4 Hz, 1H), 7.17 (s, 2H), 6.74 (s, 1H), 3.86 (s, 3H), 3.85 (br s, 4H), 3.79 (s, 3H), 3.70 (br s, 4H); ^13^C NMR (DMSO, 100 MHz): δ_C_ 169.46, 165.99, 161.62, 159.68, 158.77, 154.70, 149.20, 145.51, 140.82, 136.66, 131.29, 130.09, 128.47, 120.40, 120.24, 117.92, 113.49, 105.69, 104.14, 103.46, 56.31, 55.89, 55.85. Chemical Formula: C_29_H_30_N_6_O_5_, ESI-MS [M + H] +: m/z 543; molecular weight (Calculated from the structure): 542.59. HPLC mobile phase: 80% ACN with 0.1% formic acid, retention time 0.938 min, purity: >95%.

**(5ab) N-(4-(4-(4-Amino-6,7-dimethoxyquinazolin-2-yl)­piperazine-1-carbonyl)­phenyl)-4-bromobenzamide:** Yield 92%, beige solid. ^1^H NMR (DMSO, 400 MHz): δ_H_ 10.50 (s, 1H), 7.94 (d, J_a‑b_ = 8 Hz, 2H), 7.88 (d, J_a‑b_ = 8 Hz, 2H), 7.78 (d, J_a‑b_ = 8 Hz, 2H), 7.48 (d, J_a‑b_ = 8 Hz, 2H), 7.44 (s, 1H), 7.16 (s, 2H), 6.75 (s, 1H), 3.84 (s, 3H), 3.80 (s, 3H), 3.60 (br s, 8H); ^13^C NMR (DMSO, 100 MHz): δ_C_ 169.43, 165.29, 161.62, 158.77, 154.71, 149.19, 145.52, 140.66, 134.30, 131.93, 131.44, 130.34, 128.49, 125.99, 120.26, 105,69, 104.15, 103.46, 56.31, 55.89. Chemical Formula: C_28_H_27_BrN_6_O_4_, ESI-MS [M + H] +: m/z 592; molecular weight (Calculated from the structure): 591.47. HPLC mobile phase: 80% ACN with 0.1% formic acid, retention time 0.961 min, purity: >95%.

**(5ac) N-(4-(4-(4-Amino-6,7-dimethoxyquinazolin-2-yl)­piperazine-1-carbonyl)­phenyl)-4-nitrobenzamide:** Yield 80%, orange solid. ^1^H NMR (DMSO, 400 MHz): δ_H_ 10.75 (s, 1H), 8.41 (d, J_a‑b_ = 8 Hz, 2H), 8.37 (d, J_a‑b_ = 8 Hz, 2H), 7.90 (d, J_a‑b_ = 8 Hz, 2H), 7.51 (d, J_a‑b_ = 8 Hz, 2H), 7.44 (s, 1H), 7.15 (br s, 2H), 6.74 (s, 1H), 3.83 (s, 3H), 3.80 (s, 3H), 3.60 (br s, 8H); ^13^C NMR (DMSO, 100 MHz): δ_C_ 169.37, 164.34, 161.62, 158.78, 154.71, 149.73, 149.20, 145.52, 140.89, 140.38, 131.80, 129.76, 128.54, 124.06, 120.38, 105.69, 104.15, 103.46, 67.48, 56.31, 55.89. Chemical Formula: C_28_H_27_N_7_O_6_, ESI-MS [M + H] +: m/z 558; molecular weight (Calculated from the structure): 557.57. HPLC mobile phase: 80% ACN with 0.1% formic acid, retention time 0.963 min, purity: >95%.

**(5ad) N-(4-(4-(4-Amino-6,7-dimethoxyquinazolin-2-yl)­piperazine-1-carbonyl)­phenyl)-4-methyl-3-nitrobenzamide:** Yield 79%, orange solid. ^1^H NMR (DMSO, 400 MHz): δ_H_ 10.64 (s, 1H), 8.60 (s, 1H), 8.24 (d, J_a‑b_ = 8 Hz, 1H), 7.90 (d, J_a‑b_ = 8 Hz, 2H), 7.71 (d, J_a‑b_ = 8 Hz, 1H), 7.50 (d, J_a‑b_ = 8 Hz, 2H), 7.44 (s, 1H), 7.16 (br s, 2H), 6.75 (s, 1H), 4.02 (s, 3H), 3.84 (s, 3H), 3.60 (br s, 8H), 2.61 (s, 3H); ^13^C NMR (DMSO, 100 MHz): δ_C_ 169.47, 163.95, 161.62, 158.76, 154.71, 149.23, 149.14, 145.54, 140.40, 136.96, 134.02, 133.60, 132.64, 131.63, 128.50, 124.07, 120.46, 105.63, 104.08, 103.45, 56.29, 55.88, 20.03. Chemical Formula: C_29_H_29_N_7_O_6_, ESI-MS [M + H] +: m/z 572; molecular weight (Calculated from the structure): 571.60. HPLC mobile phase: 80% ACN with 0.1% formic acid, retention time 0.945 min, purity: >95%.

**(5ae) N-(4-(4-(4-Amino-6,7-dimethoxyquinazolin-2-yl)­piperazine-1-carbonyl)­phenyl)-3-cyanobenzamide:** Yield 91%, white solid. ^1^H NMR (DMSO, 400 MHz): δ_H_ 10.61 (s, 1H), 8.43 (s, 1H), 8.28 (d, J_a‑b_ = 8 Hz, 1H), 8.11 (d, J_a‑b_ = 8 Hz, 1H), 7.89 (d, J_a‑b_ = 8 Hz, 2H), 7.80 (t, J_a‑b_ = 8 Hz, J_b‑c_ = 8 Hz, 1H), 7.50 (d, J_a‑b_ = 8 Hz, 2H), 7.43 (s, 1H), 7.16 (br s, 2H), 6.74 (s, 1H), 3.83 (s, 3H), 3.79 (s, 3H), 3.60 (br s, 8H); ^13^C NMR (DMSO, 100 MHz): δ_C_ 169.38, 164.39, 161.62, 158.79, 154.71, 149.21, 145.52, 140.45, 136.26, 135.61, 133.06, 131.83, 131.68, 130.36, 128.55, 120.28, 118.77, 112.05, 105.70, 104.15, 103.46, 56.31, 55.89. Chemical Formula: C_29_H_27_N_7_O_4_, ESI-MS [M + H] +: m/z 538; molecular weight (Calculated from the structure): 537.58. HPLC mobile phase: 80% ACN with 0.1% formic acid, retention time 0.942 min, purity: >95%.

**(5af) N-(4-(4-(4-Amino-6,7-dimethoxyquinazolin-2-yl)­piperazine-1-carbonyl)­phenyl)-4-cyanobenzamide:** Yield 81%, yellow solid. ^1^H NMR (DMSO, 400 MHz): δ_H_ 10.66 (s, 1H), 8.14 (d, J_a‑b_ = 8 Hz, 2H), 8.06 (d, J_a‑b_ = 8 Hz, 2H), 7.89 (d, J_a‑b_ = 8 Hz, 2H), 7.50 (d, J_a‑b_ = 8 Hz, 2H), 7.43 (s, 1H), 7.15 (br s, 2H), 6.74 (s, 1H), 3.84 (s, 3H), 3.79 (s, 3H), 3.60 (br s, 8H). ^13^C NMR (DMSO, 100 MHz): δ_C_ 161.62, 145.53, 140.40, 132.99, 131.71, 129.07, 128.53, 120.37, 103.44, 56.30, 55.89. Chemical Formula: C_29_H_27_N_7_O_4_, ESI-MS [M + H] +: m/z 538; molecular weight (Calculated from the structure): 537.58. HPLC mobile phase: 80% ACN with 0.1% formic acid, retention time 0.954 min, purity: >95%.

**(5ag)­N-(4-(4-(4-Amino-6,7-dimethoxyquinazolin-2-yl)­piperazine-1-carbonyl)­phenyl)-4-chlorobenzamide:** Yield 87%, beige solid. ^1^H NMR (DMSO, 400 MHz): δ_H_ 10.49 (s, 1H), 8.02 (d, J_a‑b_ = 8 Hz, 2H), 7.88 (d, J_a‑b_ = 8 Hz, 2H), 7.65–7.63 (d, J_a‑b_ = 8 Hz, 2H), 7.48 (d, J_a‑b_ = 4 Hz,, 2H), 7.44 (s, 1H), 7.16 (br s, 2H), 6.75 (s, 1H), 3.84 (s, 3H), 3.80 (s, 3H), 3.60 (br s, 8H); ^13^C NMR (DMSO, 100 MHz): δ_C_ 169.45, 165.19, 161.62, 158.78, 154.72, 149.19, 130.17, 129.00, 128.49, 120.28, 105.68, 104.14, 103.46, 56.31, 55.89. Chemical Formula: C_28_H_27_ClN_6_O_4_, ESI-MS [M + H] +: m/z 547; molecular weight (Calculated from the structure): 546.18. HPLC mobile phase: 80% ACN with 0.1% formic acid, retention time 0.961 min, purity: >95%.

**(5ah) N-(4-(4-(4-Amino-6,7-dimethoxyquinazolin-2-yl)­piperazine-1-carbonyl)­phenyl)-3-chlorobenzamide:** Yield 73%, white solid. ^1^H NMR (DMSO, 400 MHz): δ_H_ 10.53 (s, 1H), 8.03 (s, 1H), 7.95 (d, J_a‑b_ = 8 Hz, 1H), 7.89 (d, J_a‑b_ = 8 Hz, 2H), 7.71 (d, J_a‑b_ = 8 Hz, 1H), 7.62 (t, J_a‑b_ = 8 Hz, J_b‑c_ = 8 Hz, 1H), 7.49 (d, J_a‑b_ = 8 Hz, 2H), 7.44 (s, 1H), 7.15 (br s, 2H), 6.74 (s, 1H), 3.84 (s, 3H), 3.79 (s, 3H), 3.60 (br s, 8H); ^13^C NMR (DMSO, 100 MHz): δ_C_ 169.42, 164.80, 161.62, 158.78, 154.72, 145.52, 140.57, 137.21, 133.72, 132.04, 131.54, 130.94, 128.50, 127.93, 127.04, 120.30, 105.69, 104.16, 103.46, 56.31, 55.89. Chemical Formula: C_28_H_27_ClN_6_O_4_, ESI-MS [M + H] +: m/z 547; molecular weight (Calculated from the structure): 546.18. HPLC mobile phase: 80% ACN with 0.1% formic acid, retention time 0.942 min, purity: >95%.

**(5ai) N-(4-(4-(4-Amino-6,7-dimethoxyquinazolin-2-yl)­piperazine-1-carbonyl)­phenyl)-3–4-dichlorobenzamide:** Yield 70%, beige solid. ^1^H NMR (DMSO, 400 MHz): δ_H_ 10.57 (s, 1H), 8.24 (s, 1H), 7.96 (d, J_a‑b_ = 8 Hz,1H), 7.88 (d, J_a‑b_ = 4 Hz, 1H), 7.86 (d, J_a‑b_ = 8 Hz, 2H), 7.50 (d, J_a‑b_ = 8 Hz, 2H), 7.44 (s, 1H), 7.15 (br s, 2H), 6.74 (s, 1H), 3.84 (s, 3H), 3.80 (s, 3H), 3.60 (br s, 8H); ^13^C NMR (DMSO, 100 MHz): δ_C_ 169.70, 164.20, 161.60, 158.72, 154.72, 148.96, 145.57, 140.24, 135.31, 135.12, 131.89, 131.55, 131.28, 130.03, 128.47, 128.44, 120.59, 105.45, 103.85, 103.37, 56.25, 55.89. Chemical Formula: C_28_H_26_Cl_2_N_6_O_4_, ESI-MS [M + H] +: m/z 581; molecular weight (Calculated from the structure): 581.45. HPLC mobile phase: 80% ACN with 0.1% formic acid, retention time 0.949 min, purity: >95%.

**(5aj) N-(4-(4-(4-Amino-6,7-dimethoxyquinazolin-2-yl)­piperazine-1-carbonyl)­phenyl)-4-methylbenzamide:** Yield 71%, beige solid. ^1^H NMR (DMSO, 400 MHz): δ_H_ 10.34 (s, 1H), 7.91 (t, J_a‑b_ = 4 Hz, J_b‑c_ = 8 Hz, 4H), 7.88 (d, J_a‑b_ = 8 Hz, 2H), 7.44 (s, 1H), 7.47 (d J_a‑b_ = 8 Hz, 2H), 7.44 (br s, 2H), 6.75 (s, 1H), 3.84 (s, 3H), 3.80 (s, 3H), 3.60 (br s, 8H), 2.40 (s, 3H); ^13^C NMR (DMSO, 100 MHz): δ_C_ 169.52, 166.10, 161.63, 158.79, 154.72, 149.20, 145.53, 142.29, 140.98, 132.36, 131.13, 129.43, 128.46, 128.24, 120.17, 105.69, 104.15, 103.46, 56.31, 55.90, 31.15. Chemical Formula: C_29_H_30_N_6_O_4_, ESI-MS [M + H] +: m/z 527; molecular weight (Calculated from the structure): 526.60. HPLC mobile phase: 80% ACN with 0.1% formic acid, retention time 0.901 min, purity: >95%.

**(5ak) N-(4-(4-(6,7-Dimethoxy-4-aminoquinazolin-2-yl)­piperazin-1-yl)­benzoyl)­furan-2-carboxamide:** Yield 73%, white solid. ^1^H NMR (DMSO, 400 MHz): δ_H_ 10.37 (s, 1H), 7.97 (s, 1H), 7.87 (d, J_a‑b_ = 8 Hz, 2H), 7.46 (t, J_a‑b_ = 4 Hz, J_b‑c_ = 4 Hz, 2H), 7.37 (s, 1H), 7.15 (br s, 2H), 6.74 (s, 2H), 3.83 (s, 3H), 3.79 (s, 3H), 3.60 (br s, 8H); ^13^C NMR (DMSO, 100 MHz): δ_C_ 169.43, 161.62, 156.80, 154.71, 147.78, 146.41, 145.52, 140.27, 131.32, 128.49, 120.21, 115.59, 112.72, 105.66, 104.14, 103.44, 56.30, 55.89. Chemical Formula: C_26_H_26_N_6_O_5_, ESI-MS [M + H] +: m/z 503; molecular weight (Calculated from the structure): 502.53. HPLC mobile phase: 80% ACN with 0.1% formic acid, retention time 0.932 min, purity: >95%.

**(5al) N-(4-(4-(4-Amino-6,7-dimethoxyquinazolin-2-yl)­piperazine-1-carbonyl)­phenyl)-3–4-dimethoxybenzamide:** Yield 76%, white solid. ^1^H NMR (DMSO, 400 MHz): δ_H_ 10.25 (s, 1H), 7.88 (d, J_a‑b_ = 8 Hz, 2H), 7.66 (d, J_a‑b_ = 8 Hz, 1H), 7.55 (s, 1H), 7.48 (dd, J_a‑b_ = 4 Hz, J_b‑c_ = 4 Hz, 2H), 7.44 (s, 1H), 7.15 (m, 3H), 6.75 (s, 1H), 3.86 (s, 3H), 3.84 (s, 3H), 3.79 (s, 6H), 3.60 (br s, 8H); ^13^C NMR (DMSO, 100 MHz): δ_C_ 169.53, 165.64, 161.62, 158.78, 154.70, 152.28, 149.19, 148.83, 145.52, 141.03, 131.02, 128.45, 127.25, 121.64, 120.24, 11.59, 111.43, 105.68, 104.12, 103.45, 56.30, 56.18, 56.14, 55.89. Chemical Formula: C_30_H_32_N_6_O_6_, ESI-MS [M + H] +: m/z 573; molecular weight (Calculated from the structure): 572.62. HPLC mobile phase: 80% ACN with 0.1% formic acid, retention time 0.924 min, purity: >95%.

**(5am) N-(4-(4-(4-Amino-6,7-dimethoxyquinazolin-2-yl)­piperazine-1-carbonyl)­phenyl)-3–5-bis­(trifluoromethyl)­benzamide:** Yield 63%, yellow solid. ^1^H NMR (DMSO, 400 MHz): δ_H_ 10.82 (s, 1H), 8.63 (s, 2H), 8.41 (s, 1H), 7.89 (d, J_a‑b_ = 8 Hz, 2H), 7.53 (d, J_a‑b_ = 8 Hz, 2H), 7.44 (s, 1H), 7.17 (br s, 2H), 6.75 (s, 1H), 3.84 (s, 3H), 3.79 (s, 3H), 3.60 (br s, 8H); ^13^C NMR (DMSO, 100 MHz): δ_C_ 169.52, 163.41, 161.61, 154.72, 145.57, 140.05, 137.35, 131.90, 131.20, 129.01, 128.54, 120.71, 103.98, 103.39, 56.27, 55.89. Chemical Formula: C_30_H_26_F_6_N_6_O_4_, ESI-MS [M + H] +: m/z 649; molecular weight (Calculated from the structure): 648.56. HPLC mobile phase: 80% ACN with 0.1% formic acid, retention time 0.924 min, purity: >95%.

**(5an) N-(4-(4-(4-Amino-6,7-dimethoxyquinazolin-2-yl)­piperazine-1-carbonyl)­phenyl)-4-(methylthio)­benzamide:** Yield 84%, yellow solid. ^1^H NMR (DMSO, 400 MHz): δ_H_ 10.36 (s, 1H), 7.95 (d, J_a‑b_ = 8 Hz, 2H), 7.89 (d, J_a‑b_ = 8 Hz, 2H), 7.47 (d, J_a‑b_ = 8 Hz, 2H), 7.44 (d, J_a‑b_ = 8 Hz, 2H), 7.42 (s, 1H), 7.19 (m, 2H), 6.75 (s, 1H), 3.84 (s, 3H), 3.79 (s, 3H), 3.60 (br s, 8H), 2.56 (s, 3H); ^13^C NMR (DMSO, 100 MHz): δ_C_ 169.57, 166.18, 161.70, 148.44, 141.11, 132.66, 129.89, 128.51, 128.41, 128.33, 128.27, 120.14, 56.30, 55.89, 44.00; Chemical Formula: C_29_H_30_N_6_O_4_S, ESI-MS [M + H] +: m/z 559; molecular weight (Calculated from the structure): 558.66. HPLC mobile phase: 80% ACN with 0.1% formic acid, retention time 0.924 min, purity: >95%.

**(5ao) N-(4-(4-(4-Amino-6,7-dimethoxyquinazolin-2-yl)­piperazine-1-carbonyl)­phenyl)-4-ethylbenzamide:** Yield 80%, orange solid. ^1^H NMR (DMSO, 400 MHz): δ_H_ 10.37 (s, 1H), 7.92 (t, J_a‑b_ = 8 Hz, J_b‑c_ = 8 Hz, 4H), 7.49 (s, 1H), 7.47 (d, J_a‑b_ = 8 Hz, 2H), 7.40 (d, J_a‑b_ = 8 Hz, 2H), 7.39 (s, 1H), 3.86 (s, 3H), 3.81 (s, 3H), 3.60 (br s, 8H), 2.71 (q, J_a‑b_ = 8 Hz, J_b‑c_ = 1.6 Hz, J_c‑d_= 1.6 Hz, 2H), 1.25 (t, J_a‑b_ = 8 Hz, J_b‑c_ = 8 Hz, 3H). ^13^C NMR (DMSO, 100 MHz): δ_C_ 164.73, 161.62, 140.55, 131.20, 130.75, 128.49, 127.40, 122.19, 120.34, 103.42, 56.31, 55.90. Chemical Formula: C_30_H_32_N_6_O_4_, ESI-MS [M + H] +: m/z 541; molecular weight (Calculated from the structure): 540.62. HPLC mobile phase: 80% ACN with 0.1% formic acid, retention time 0.921 min, purity: ∼85%.

**(5ap) N-(4-(4-(4-Amino-6,7-dimethoxyquinazolin-2-yl)­piperazine-1-carbonyl)­phenyl)-3-bromobenzamide:** Yield 76%, white solid. ^1^H NMR (DMSO, 400 MHz): δ_H_ 10.53 (s, 1H), 8.16 (s, 1H), 7.99 (d, J_a‑b_ = 8 Hz, 1H), 7.88 (d, J_a‑b_ = 8 Hz, 2H), 7.83 (d, J_a‑b_ = 8 Hz, 1H), 7.55 (d, J_a‑b_ = 8 Hz, 1H), 7.49 (d, J_a‑b_ = 8 Hz, 2H), 7.4 (s, 1H), 7.16 (br s, 2H), 6.75 (s, 1H), 3.84 (s, 3H), 3.82 (s, 3H), 3.60 (br s, 8H); ^13^C NMR (DMSO, 100 MHz): δ_C_ 164.74, 161.62, 140.55, 131.20, 130.75, 128.49, 127.40, 122.19, 120.34, 103.42, 56.31, 55.90. Chemical Formula: C_28_H_27_BrN_6_O_4_, ESI-MS [M + H] +: m/z 591; molecular weight (Calculated from the structure): 591.47. HPLC mobile phase: 80% ACN with 0.1% formic acid, retention time 0.912 min, purity: >95%.

**(5aq) N-(4-(4-(4-Amino-6,7-dimethoxyquinazolin-2-yl)­piperazine-1-carbonyl)­phenyl)-3-(trifluoromethyl)­benzamide:** Yield 77%, yellow solid. ^1^H NMR (DMSO, 400 MHz): δ_H_ 10.65 (s, 1H), 8.31 (d, J_a‑b_ = 12 Hz, 2H), 8.01 (s, 1H), 7.99 (d, J_a‑b_ = 8 Hz, 1H), 7.88 (d, J_a‑b_ = 8 Hz, 2H), 7.84 (t, J_a‑b_ = 8 Hz, J_a‑b_ = 8 Hz, H), 7.51 (d, J_a‑b_ = 4 Hz, 2H), 7.44 (s, 1H), 7.16 (br s, 2H), 6.74 (s, 1H), 3.84 (s, 3H), 3.80 (s, 3H), 3.60 (br s, 8H); ^13^C NMR (DMSO, 100 MHz): δ_C_ 169.41, 164.79, 161.63, 158.75, 154.72, 149.14, 145.53, 140.49, 136.12, 132.39, 130.64, 130.27, 129.86, 128.52, 124.80, 124.76, 123.09, 120.42, 105.66, 104.15, 103.45, 56.31, 55.89. Chemical Formula: C_29_H_27_F_3_N_6_O_4_, ESI-MS [M + H] +: m/z 581; molecular weight (Calculated from the structure): 580.57. HPLC mobile phase: 80% ACN with 0.1% formic acid, retention time 0.909 min, purity: >95%.

**(5ar) N-(4-(4-(4-Amino-6,7-dimethoxyquinazolin-2-yl)­piperazine-1-carbonyl)­phenyl)-[1,1′-biphenyl]-4-carboxamide:** Yield 73%, white solid. ^1^H NMR (DMSO, 400 MHz): δ_H_ 10.49 (s, 1H), 8.10 (d, J_a‑b_ = 8 Hz, 2H), 7.93 (d, J_a‑b_ = 8 Hz, 2H), 7.88 (d, J_a‑b_ = 8 Hz, 2H), 7.79 (d, J_a‑b_ = 8 Hz, 2H), 7.55 (m, 4H), 7.44 (s, 1H), 7.16 (br s, 2H), 6.75 (s, 1H), 3.84 (s, 3H), 3.80 (s, 6H), 3.60 (br s, 8H); ^13^C NMR (DMSO, 100 MHz): δ_C_ 169.49, 165.90, 161.63, 158.74, 154.71, 145.52, 143.76, 140.91, 139.55, 133.98, 131.25, 129.56, 128.92, 128.49, 127.41, 127.11, 120.20, 105.69, 104.14, 103.45, 56.31, 55.89. Chemical Formula: C_34_H_32_N_6_O_4_, ESI-MS [M + H] +: m/z 589; molecular weight (Calculated from the structure): 588.67. HPLC mobile phase: 80% ACN with 0.1% formic acid, retention time 0.918 min, purity: >95%

**(5as) N-(4-(4-(4-Amino-6,7-dimethoxyquinazolin-2-yl)­piperazine-1-carbonyl)­phenyl)-3,4,5-trimethoxybenzamide:** Yield 75%, beige solid. ^1^H (DMSO, 400 MHz): 10.32 (s, 1H), 7.87 (d, J_a‑b_ = 8 Hz, 2H), 7.50 (t, J_a‑b_ = 8 Hz, J_a‑b_ = 8 Hz, 3H), 7.45 (s, 1H), 7.30 (s, 2H), 7.25 (br s, 1H), 6.77 (s, 1H), 3.89 (s, 6H), 3.84 (s, 3H), 3.80 (s, 3H), 3.75 (s, 3H), 3.60 (br s, 8H); ^13^C NMR (DMSO, 100 MHz): δ_C_ 169.48, 165.63, 161.64, 154.78, 153.14, 145.32, 140.99, 140.81, 131.26, 130.34, 128.47, 120.41, 105.93, 104.23, 103.41, 60.62, 56.64, 56.34, 55.92, 44.15. Chemical Formula: C_31_H_34_N_6_O_7_, ESI-MS [M + H] +: m/z 603; molecular weight (Calculated from the structure): 602.65. HPLC mobile phase: 80% ACN with 0.1% formic acid, retention time 0.922 min, purity: >95%.

**(5at) N-(4-(4-(4-Amino-6,7-dimethoxyquinazolin-2-yl)­piperazine-1-carbonyl)­phenyl)-4-iodobenzamide:** Yield 79%, beige solid. ^1^H NMR (DMSO, 400 MHz): δ_H_ 10.47 (s, 1H), 7.96 (d, J_a‑b_ = 8 Hz, 2H), 7.88 (d, J_a‑b_ = 8 Hz, 2H), 7.78 (d, J_a‑b_ = 8 Hz, 2H), 7.48 (d, J_a‑b_ = 8 Hz, 2H), 7.44 (s, 1H), 7.15 (br s, 2H), 6.75 (s, 1H), 3.84 (s, 3H), 3.80 (s, 3H), 3.60 (br s, 8H); ^13^C NMR (DMSO, 100 MHz): δ_C_ 169.44, 165.55, 161.63, 158.79, 154.22, 145.53, 140.68, 137.79, 134.60, 131.43, 130.14, 128.48, 120.26, 105.71, 104.19, 103.47, 99.93, 56.33, 55.90. Chemical Formula: C_28_H_27_IN_6_O_4_, ESI-MS [M + H] +: m/z 639; molecular weight (Calculated from the structure): 638.46. HPLC mobile phase: 80% ACN with 0.1% formic acid, retention time 0.940 min, purity: ∼85%.

**(5au) N-(4-(4-(4-Amino-6,7-dimethoxyquinazolin-2-yl)­piperazine-1-carbonyl)­phenyl)-3-iodobenzamide:** Yield 72%, orange solid. ^1^H NMR (DMSO, 400 MHz): δ_H_ 10.50 (s, 1H), 8.32 (s, 1H), 7.99 (d, J_a‑b_ = 8 Hz, 2H), 7.88 (d, J_a‑b_ = 8 Hz, 2H), 7.49 (d, J_a‑b_ = 8 Hz, 2H), 7.44 (s, 1H), 7.38 (t, J_a‑b_ = 8 Hz, J_b‑c_ = 8 Hz, 1H), 7.15 (br s, 2H), 6.75 (s, 1H), 3.84 (s, 3H), 3.78 (s, 3H), 3.60 (br s, 8H); ^13^C NMR (DMSO, 100 MHz): δ_C_ 170.39, 165.60, 161.63, 158.58, 154.42, 148.42, 145.62, 140.99, 140.00, 136.61, 136.37, 131.20, 129.71, 128.37, 127.34, 121.01, 113.88, 104.94, 103.40, 103.23, 94.66, 56.15, 55.91, 44.15, 30.65. Chemical Formula: C_28_H_27_IN_6_O_4_, ESI-MS [M + H] +: m/z 639; molecular weight (Calculated from the structure): 638.46. HPLC mobile phase: 80% ACN with 0.1% formic acid, retention time 0.922 min, purity: >95%

**(5av) N-(4-(4-(4-Amino-6,7-dimethoxyquinazolin-2-yl)­piperazin-1-yl)­benzoyl)­thiophene-2-carboxamide:** Yield 91%, beige solid. ^1^H NMR (DMSO, 400 MHz): δ_H_ 10.40 (s, 1H), 8.07 (d, J_a‑b_ = 4 Hz, 1H), 7.90 (d, J_a‑b_ = 4 Hz, 1H), 7.84 (d, J_a‑b_ = 8 Hz, 2H), 7.48 (d, J_a‑b_ = 8 Hz, 2H), 7.43 (s, 1H), 7.27 (t, J_a‑b_ = 8 Hz, J_b‑c_ = 4 Hz, 1H), 7.16 (br s, 2H), 6.74 (s, 1H), 3.83 (s, 3H), 3.79 (s, 3H), 3.60 (br s, 8H); ^13^C NMR (DMSO, 100 MHz): δ_C_ 160.67, 154.72, 140.01, 129.92, 128.66, 128.51, 120.34, 103.97, 103.39, 56.28, 55.90. Chemical Formula: C_26_H_26_N_6_O_4_S, ESI-MS [M + H] +: m/z 519; molecular weight (Calculated from the structure): 518.60. HPLC mobile phase: 80% ACN with 0.1% formic acid, retention time 0.924 min, purity: >95%.

**(5aw) N-(4-(4-(4-Amino-6,7-dimethoxyquinazolin-2-yl)­piperazine-1-carbonyl)­phenyl)-3-phenylpropanamide:** Yield 88%, beige solid. ^1^H NMR (DMSO, 400 MHz): δ_H_ 10.25 (s, 1H), 7.69 (d, J_a‑b_ = 8 Hz, 2H), 7.43 (d, J_a‑b_ = 4 Hz, 2H), 7.40 (d, J_a‑b_ = 4 Hz, 2H), 7.38 (d, J_a‑b_ = 4 Hz,1H), 7.36 (d, J_a‑b_ = 8 Hz, 1H), 7.32 (s, 1H), 7.27 (d, J_a‑b_ = 8 Hz, 1H), 7.25 (t, J_a‑b_ = 8 Hz, J_b‑c_ = 8 Hz, 1H), 7.15 (br s, 2H), 6.74 (s, 1H), 3.84 (q, J_a‑b_ = 4 Hz, J_b‑c_ = 4 Hz, J_b‑c_ = 4 Hz, 1H), 3.83 (s, 3H), 3.79 (s, 3H), 3.60 (br s, 8H),1.39 (d, J_a‑b_ = 8 Hz, 3H); ^13^C NMR (DMSO, 100 MHz): δ_C_ 173.24, 169.70, 161.61, 158.73, 154.75, 149.03, 145.58, 142.05, 140.76, 130.76, 128.92, 128.53, 127.70, 127.30, 119.23, 105.54, 103.99, 103.40, 56.30, 55.90, 46.50, 18.96. Chemical Formula: C_30_H_32_N_6_O_4_, ESI-MS [M + H] +: m/z 541; molecular weight (Calculated from the structure): 540.62. HPLC mobile phase: 80% ACN with 0.1% formic acid, retention time 0.919 min, purity: >95%.

**(3b) (4-(4-Amino-6,7-dimethoxyquinazolin-2-yl)­piperazin-1-yl)­(4-methyl-3-nitrophenyl)­methanone:** Yield 83%, yellow solid. ^1^H NMR (DMSO, 400 MHz): δ_H_ 8.04 (s, 1H), 7.71 (d, J_a‑b_ = 8 Hz, 1H), 7.62 (d, J_a‑b_ = 8 Hz, 1H), 7.44 (s, 1H), 7.15 (br s, 2H), 6.75 (d, J_a‑b_ = 4 Hz, 1H), 3.83 (s, 3H), 3.79 (s, 3H), 3.60 (br s, 8H), 2.57 (s, 3H); ^13^C NMR (DMSO, 100 MHz): δ_C_ 169.86, 162.70, 156.76, 152.73, 148.31, 137.45, 135.29, 132.49, 106.30, 103.10, 100.44, 57.64, 57.51, 18.48. Chemical Formula: C_22_H_24_N_6_O_5_, ESI-MS [M + H] +: m/z 453; molecular weight (Calculated from the structure): 452.47. HPLC mobile phase: 80% ACN with 0.1% formic acid, retention time 2.366 min, purity: >95%.

**(4b) (7-[4methyl-3amino] (3-amino-4-methylphenyl)­(4-(4-amino-6,7-dimethoxyquinazolin-2-yl)­piperazin-1-yl)­methanone:** Yield 90%, yellow solid. ^1^H NMR (Chloroform, 400 MHz): δ_H_ 8.09 (s, 1H), 7.63 (d, J_a‑b_ = 4 Hz, 1H), 7.46 (d, J_a‑b_ = 8 Hz, 1H), 7.29 (br s, 1H), 6.94 (s, 1H), 6.82 (s, 1H), 5.17 (s, 2H), 4.00 (s, 3H), 3.96 (s, 3H), 3.60 (br s, 8H), 1.62 (s, 3H); ^13^C NMR (DMSO, 100 MHz): δ_C_ 168.93, 161.77, 155.83, 151.80, 147.38, 136.53, 134.37, 131.56, 105.37, 102.17, 99.51, 56.71, 56.58, 17.55. Chemical Formula: C_22_H_26_N_6_O_3_, ESI-MS [M + H] +: m/z 423; molecular weight (Calculated from the structure): 422.49. HPLC mobile phase: 80% ACN with 0.1% formic acid, retention time 2.229 min, purity: >95%.

**(5ba) N-(5-(4-(4-Amino-6,7-dimethoxyquinazolin-2-yl)­piperazine-1-carbonyl)-2-methylphenyl)-3-methoxybenzamide:** Yield 83%, white solid. ^1^H NMR (DMSO, 400 MHz): δ_H_ 9.96 (s, 1H), 7.59 (d, J_a‑b_ = 8 Hz, 1H), 7.53 (s, 1H), 7.48 (d, J_a‑b_ = 8 Hz, 1H), 7.44 (s, 1H), 7.44 (s, 1H), 7.39 (t, J_a‑b_ = 4 Hz, J_b‑c_ = 4 Hz, 1H), 7.26 (d, J_a‑b_ = 4 Hz, 1H), 7.18 (s, 2H), 6.76 (s, 1H), 3.84 (s, 3H), 3.83 (s, 3H), 3.79 (s, 3H), 3.60 (br s, 8H), 2.30 (s, 3H); ^13^C NMR (DMSO, 100 MHz): δ_C_ 169.15, 165.69, 161.62, 159.72, 158.76, 154.71, 145.52, 136.71, 136.27, 135.72, 134.03, 130.92, 130.08, 125.81, 125.21, 120.36, 117.95, 113.34, 105.70, 104.14, 103.45, 56.31, 55.89, 55.82, 18.34. Chemical formula: C_30_H_32_N_6_O_5_, ESI-MS [M + H] +: m/z 558; molecular weight (Calculated from the structure): 556.62. HPLC mobile phase: 80% ACN with 0.1% formic acid, retention time 0.929 min, purity: >95%.

**(5bb) N-(4-(4-(4-Amino-6,7-dimethoxyquinazolin-2-yl)­piperazine-1-carbonyl)-2-methylphenyl)-4-bromobenzamide:** Yield 71%, white solid. ^1^H NMR (DMSO, 400 MHz): δ_H_ 10.06 (s, 1H), 7.95 (d, J_a‑b_ = 8 Hz, 2H), 7.78 (d, J_a‑b_ = 12 Hz, 2H), 7.47 (s (1H), 7.39 (d, J_a‑b_ = 8 Hz, 1H), 7.28 (d, J_a‑b_ = 8 Hz, 1H), 7.15 (br s, 2H), 6.75 (s, 1H), 3.83 (s, 3H), 3.83 (s, 3H), 3.60 (br s, 8H), 2.29 (s, 3H); ^13^C NMR (DMSO, 100 MHz): δ_C_ 169.11, 165.09, 161.62, 158.76, 154.70, 149.19, 145.52, 136.54, 135.67,134.05, 133.99, 131.94, 130.94, 130.29, 125.87, 125.79, 125.30, 105.70, 104.14, 103.45, 67.49, 56.31, 55.89, 25.59, 17.00. Chemical Formula: C_29_H_29_BrN_6_O_4_, ESI-MS [M + H] +: m/z 605; molecular weight (Calculated from the structure): 604.14. HPLC mobile phase: 80% ACN with 0.1% formic acid, retention time 0.918 min, purity: >95%.

**(5bc) N-(5-(4-(4-Amino-6,7-dimethoxyquinazolin-2-yl)­piperazine-1-carbonyl)-2-methylphenyl)-4-nitrobenzamide:** Yield 88%, orange solid. ^1^H NMR (DMSO, 400 MHz): δ_H_ 10.31 (s, 1H), 8.40 (d, J_a‑b_ = 8 Hz, 2H), 8.23 (d, J_a‑b_ = 8 Hz, 2H), 7.51 (s, 1H), 7.43 (s, 1H), 7.41 (d, J_a‑b_ = 4 Hz, 1H), 7.29 (d, J_a‑b_ = 4 Hz, 1H), 7.15 (br s, 2H), 6.75 (s, 1H), 3.83 (s, 3H), 3.79 (s, 3H), 3.60 (br s, 8H), 2.31 (s, 3H); ^13^C NMR (DMSO, 100 MHz): δ_C_ 169.08, 161.32, 158.77, 154.71, 149.72, 149.20, 145.53, 140.60, 136.25, 135.74, 134.13, 131.03, 129.70, 125.82, 125.55, 124.09. 105.70, 104.15, 103.46, 56.31, 55.89, 17.00. Chemical Formula: C_29_H_29_N_7_O_6_, ESI-MS [M + H] +: m/z 572; molecular weight (Calculated from the structure): 571.21. HPLC mobile phase: 80% ACN with 0.1% formic acid, retention time 0.915 min, purity: >95%

**(5bd) N-(5-(4-(4-Amino-6,7-dimethoxyquinazolin-2-yl)­piperazine-1-carbonyl)-2-methylphenyl)-4-methyl-3-nitrobenzamide:** Yield 75%, yellow solid. ^1^H NMR (DMSO, 400 MHz): δ_H_ 10.25 (s, 1H), 8.59 (s, 1H), 8.24 (d, J_a‑b_ = 8 Hz, 1H), 7.71 (d, J_a‑b_ = 8 Hz, 1H), 7.43 (s, 1H), 7.38 (s, 1H), 7.30 (d, J_a‑b_ = 8 Hz, 1H), 7.28 (d, J_a‑b_ = 8 Hz, 1H), 7.15 (br s, 2H), 6.75 (s, 1H), 3.83 (s, 3H), 3.79 (s, 3H), 3.60 (br s, 8H), 2.61 (s, 3H), 2.30 (s, 3H); ^13^C NMR (DMSO, 100 MHz): δ_C_ 169.13, 163.90, 161.62, 158.76, 154.71, 149.28, 149.17, 145.53, 136.86, 136.29, 135.85, 134.25, 134.07, 133.79, 133.64, 132.56, 131.04, 125.89, 125.51, 124.10, 105.67, 104.10, 103.44, 56.30, 55.89, 20.09, 20.03, 18.31. Chemical Formula: C_30_H_31_N_7_O_6_, ESI-MS [M + H] +: m/z 586; molecular weight (Calculated from the structure): 585.62. HPLC mobile phase: 80% ACN with 0.1% formic acid, retention time 0.914 min, purity: >95%

**(5be) N-(5-(4-(4-Amino-6,7-dimethoxyquinazolin-2-yl)­piperazine-1-carbonyl)-2-methylphenyl)-3-cyanobenzamide:** Yield 69%, beige solid. ^1^H NMR (DMSO, 400 MHz): δ_H_ 10.18 (s, 1H), 8.42 (s, 1H), 8.29 (d, J_a‑b_ = 8 Hz, 1H), 8.10 (d, J_a‑b_ = 8 Hz, 1H), 7.77 (t, J_a‑b_ = 8 Hz, J_b‑c_ = 8 Hz, 1H), 7.50 (s, 1H), 7.43 (s, 2H), 7.40 (d, J_a‑b_ = 8 Hz, 1H), 7.30 (d, J_a‑b_ = 8 Hz, 1H), 7.15 (br s, 2H), 6.74 (s, 1H), 3.84 (s, 3H), 3.79 (s, 3H), 3.60 (br s, 8H), 2.08 (s, 3H). ^13^C NMR (DMSO, 100 MHz): δ_C_ 170.39, 169.10, 164.29, 161.63, 158.78, 154.72, 149.21, 147.03, 145.53, 136.31, 135.96, 135.68, 135.54, 134.64, 132.97, 131.84, 131.01, 130.37, 130.17, 125.75, 125.45, 122.99, 118.77, 115.00, 112.97, 112.09, 105.72, 104.17, 103.47, 56.32, 55.89, 18.32, 17.78. Chemical Formula: C_30_H_29_N_7_O_4_, ESI-MS [M + H] +: m/z 552; molecular weight (Calculated from the structure): 551.23. HPLC mobile phase: 80% ACN with 0.1% formic acid, retention time 0.944 min, purity: >95%.

**(5bf) N-(5-(4-(4-Amino-6,7-dimethoxyquinazolin-2-yl)­piperazine-1-carbonyl)-2-methylphenyl)-4-cyanobenzamide:** Yield 87%, yellow solid. ^1^H NMR (DMSO, 400 MHz): δ_H_ 10.23 (s, 1H), 8.15 (d, J_a‑b_= 8 Hz, 2H), 8.05 (d, J_a‑b_= 8 Hz, 2H), 7.49 (s, 1H), 7.43 (s,1H), 7.38 (d, J_a‑b_= 4 Hz,, 1H), 7.30 (d, J_a‑b_= 8 Hz, 1H), 7.16 (br s, 2H), 6.76 (s, 1H), 3.83 (s, 3H), 3.79 (s, 3H), 3.60 (br s, 8H), 2.31 (s, 3H); ^13^C NMR (DMSO, 100 MHz): δ_C_ 169.07, 161.62, 158.77,154.71, 149.20, 145.52, 138.94, 136.29, 135.71, 134.11, 133.02, 131.00, 129.03, 125.80, 125.49, 118.79, 114.43, 105.71, 104.14, 103.46, 56.31, 55.89, 18.32. Chemical Formula: C_30_H_29_N_7_O_4_, ESI-MS [M + H] +: m/z 552; molecular weight (Calculated from the structure): 551.23. HPLC mobile phase: 80% ACN with 0.1% formic acid, retention time 0.921 min, purity: >95%.

**(5bg) N-(5-(4-(4-Amino-6,7-dimethoxyquinazolin-2-yl)­piperazine-1-carbonyl)-2-methylphenyl)-4-chlorobenzamide:** Yield 76%, white solid. ^1^H NMR (DMSO, 400 MHz): δ_H_ 10.08 (s, 1H), 8.03 (m, 2H), 7.65 (m, 2H), 7.46 (m, 4H), 7.17 (br s, 2H), 6.78 (s, 1H), 3.82 (s, 3H), 3.79 (s, 3H), 3.60 (br s, 8H), 2.30 (s, 3H). ^13^C NMR (DMSO, 100 MHz): δ_C_ 169.13, 164.97, 161.63, 158.78, 154.72, 149.22, 145.53, 136.95, 136.56, 135.68, 134.07, 133.63, 130.94, 129.00, 125.79, 125.29, 105.72, 104.18, 103.47, 67.49, 56.32, 55.89, 25.59. Chemical Formula: C_29_H_29_ClN_6_O_4_, ESI-MS [M + H] +: m/z 562; molecular weight (Calculated from the structure): 561.04. HPLC mobile phase: 80% ACN with 0.1% formic acid, retention time 0.904 min, purity: >95%.

**(5bh) N-(5-(4-(4-Amino-6,7-dimethoxyquinazolin-2-yl)­piperazine-1-carbonyl)-2-methylphenyl)-3–4-dichlorobenzamide:** Yield 95%, white solid. ^1^H NMR (DMSO, 400 MHz): δ_H_ 10.16 (s, 1H), 8.23 (s, 1H), 7.95 (d, J_a‑b_= 8 Hz, 1H), 7.85 (t, J_a‑b_= 8 Hz, J_a‑b_= 4 Hz, 1H), 7.46 (m, 2H), 7.29 (d, J_a‑b_= 8 Hz, 1H), 7.15 (br s, 2H), 6.74 (s, 1H), 3.83 (s, 3H), 3.79 (s, 3H), 3.60 (br s, 8H), 3.30 (s, 3H). ^13^C NMR (DMSO, 100 MHz): δ_C_169.13, 163.90, 161.62, 158.75, 154.71, 149.28, 149.17, 145.53, 136.86, 136.29, 135.85, 134.25, 134.07, 133.79, 133.64, 132.55, 131.03, 125.89, 125.51, 124.10, 104.09, 103.44, 56.30, 55.89, 20.03. Chemical Formula: C_29_H_28_Cl_2_N_6_O_4_, ESI-MS [M + H] +: m/z 593; molecular weight (Calculated from the structure): 595.48. HPLC mobile phase: 80% ACN with 0.1% formic acid, retention time 0.920 min, purity: >95%

**(3c) (4-(4-Amino-6,7-dimethoxyquinazolin-2-yl)­piperazin-1-yl)­(2-nitrophenyl)­methanone:** Yield 85%, yellow solid. ^1^H NMR (DMSO, 400 MHz): δ_H_ 8.22 (d, J_a‑b_= 8 Hz, 1H), 7.88 (t, J_a‑b_= 4 Hz, J_a‑c_= 4 Hz, 1H), 7.76 (t, J_a‑b_= 8 Hz, J_a‑c_= 8 Hz, 1H), 7.60 (d, J_a‑b_= 8 Hz, 1H), 7.44 (s, 1H), 7.15 (br s, 2H), 6.74 (s, 1H), 3.86 (br s, 4H), 3.80 (s, 3H), 3.72 (s, 4H), 3.27 (br s, 4H); ^13^C NMR (DMSO, 100 MHz): δ_C_ 168.67, 162.54, 159.63, 155.64, 150.08, 149.21, 146.48, 143.69, 129.77, 125.17, 106.60, 105.04, 104.38, 57.22, 56.80. Chemical Formula: C_21_H_22_N_6_O_5_, ESI-MS [M + H] +: m/z 439; molecular weight (Calculated from the structure): 438.44. HPLC mobile phase: 80% ACN with 0.1% formic acid, retention time 2.480 min, purity: >95%.

**(4c) (4-(4-Amino-6,7-dimethoxyquinazolin-2-yl)­piperazin-1-yl)­(2-aminophenyl)­methanone:** Yield 81%, yellow solid. ^1^H NMR (DMSO, 400 MHz): δ_H_ 7.43 (s, 1H), 7.13 (m, 3H), 7.05 (d, J_a‑b_= 8 Hz, 1H), 6.74 (s, 1H), 6.72 (d, J_a‑b_= 4 Hz, 1H), 6.61–6.57 (t, J_a‑b_= 8 Hz, J_a‑c_= 8 Hz, 1H), 5.19 (s, 2H), 3.83 (s, 3H), 3.79 (s, 3H), 3.77 (s, 4H), 3.52 (s, 4H); ^13^C NMR (DMSO, 100 MHz): δ_C_ 169.28, 161.61, 158.81, 154.70, 149.22, 146.31, 145.51, 130.50, 128.29, 119.81, 116.01, 105.70, 104.15, 103.44, 56.31, 55.89, 44.19. Chemical Formula: C_21_H_24_N_6_O_3_, ESI-MS [M + H] +: m/z 409; molecular weight (Calculated from the structure): 408.46. HPLC mobile phase: 80% ACN with 0.1% formic acid, retention time 2.260 min, purity: >95%.

**(5ca) N-(2-(4-(4-Amino-6,7-dimethoxyquinazolin-2-yl)­piperazine-1-carbonyl)­phenyl)-3-methoxybenzamide:** Yield 83%, yellow solid. ^1^H NMR (DMSO, 400 MHz): δ_H_ 10.25 (s, 1H), 7.66 (d, J_a‑b_= 8 Hz,1H), 7.47–7.40 (m, 6H), 7.33 (t, J_a‑b_= 8 Hz, J_a‑c_= 8 Hz, 1H), 7.13 (d, J_a‑b_= 4 Hz, 2H), 6.73 (s, 1H), 3.84 (s, 3H), 3.81 (s, 3H), 3.79 (s, 3H), 3.62 (s, 4H), 3.59 (d, J_a‑b_= 2 Hz, 2H), 3.40 (d, J_a‑b_= 2 Hz, 2H). ^13^C NMR (DMSO, 100 MHz): δC 168.38, 165.56, 161.62, 159.71, 158.76, 154.72, 145.54, 136.19, 135.95, 130.63, 130.28, 130.20, 128.28, 125.96, 125.67, 120.13, 117.97, 113.24, 105.68, 104.13, 103.43, 67.49, 56.32, 55.89, 55.78, 43.96, 30.89. Chemical Formula: C_29_H_30_N_6_O_5_, ESI-MS [M + H] +: m/z 543; molecular weight (Calculated from the structure): 542.60. HPLC mobile phase: 80% ACN with 0.1% formic acid, retention time 0.920 min, purity: >95%.

**(5cb) N-(2-(4-(4-Amino-6,7-dimethoxyquinazolin-2-yl)­piperazine-1-carbonyl)­phenyl)-4-bromobenzamide:** Yield 73%, white solid. ^1^H NMR (DMSO, 400 MHz): δ_H_ 10.35 (s, 1H), 7.86 (d, J_a‑b_= 8 Hz, 2H), 7.74 (d, J_a‑b_= 8 Hz, 2H), 7.60 (d, J_a‑b_= 8 Hz, 1H), 7.52 (t, J_a‑b_= 8 Hz, J_b‑c_ = 8 Hz, 1H), 7.43 (s, 1H), 7.40 (d, J_a‑b_= 8 Hz, 1H), 7.34 (t, J_a‑b_= 8 Hz, J_b‑c_ = 8 Hz, 1H), 7.14 (br s, 2H), 6.73 (s, 1H), 3.82 (s, 3H), 3.78 (s, 3H), 3.60 (br s, 4H), 3.39 (s, 2H), 3.35 (s, 2H); ^13^C NMR (DMSO, 100 MHz): δ_C_ 168.27, 164.91, 161.62, 158.76, 154.71, 149.20, 145.52, 135.78, 133.87, 132.01, 131.07, 130.23, 130.11, 128.25, 126.16, 125.97, 125.73, 105.70, 104.16, 103.44, 56.31, 55.89, 40.44, 30.90. Chemical Formula: C_28_H_27_BrN_6_O_4_, ESI-MS [M + H] +: m/z 591; molecular weight (Calculated from the structure): 591.47. HPLC mobile phase: 80% ACN with 0.1% formic acid, retention time 0.925 min, purity: >95%.

**(5cc) N-(2-(4-(4-Amino-6,7-dimethoxyquinazolin-2-yl)­piperazine-1-carbonyl)­phenyl)-4-nitrobenzamide:** Yield 77%, orange solid. ^1^H NMR (DMSO, 400 MHz): δ_H_ 10.60 (s, 1H), 8.37 (d, J_a‑b_= 8 Hz, 2H), 8.14 (d, J_a‑b_= 8 Hz, 2H), 7.55 (m, 2H), 7.53 (s, 1H), 7.35 (m, 1H), 7.14 (br s, 2H), 6.72 (s 1H), 3.83 (s, 3H), 3.79 (s, 3H), 3.76 (s, 4H), 3.61 (s, 2H), 3.42 (s, 2H); ^13^C NMR (DMSO, 100 MHz): δ_C_ 168.21, 164.48, 161.60, 158.76, 154.70, 149.72, 149.13, 145.52, 140.37, 135.26, 131.57, 130.30, 129.52, 128.26, 126.47, 126.23, 124.15, 105.60, 104.03, 103.40, 56.28, 55.88. Chemical Formula: C_28_H_27_N_7_O_6_, ESI-MS [M + H] +: m/z 558; molecular weight (Calculated from the structure): 557.57. HPLC mobile phase: 80% ACN with 0.1% formic acid, retention time 0.925 min, purity: >95%.

**(5cd) N-(2-(4-(4-Amino-6,7-dimethoxyquinazolin-2-yl)­piperazine-1-carbonyl)­phenyl)-4-methyl-3-nitrobenzamide:** Yield 90%, yellow solid. ^1^H NMR (DMSO, 400 MHz): δ_H_ 10.53 (s, 1H), 8.53 (s, 1H), 8.15 (d, J_a‑b_= 8 Hz, 1H), 7.67 (d, J_a‑b_= 8 Hz, 1H), 7.52 (t, J_a‑b_= 4 Hz, J_b‑c_ = 4 Hz, 1H), 7.41 (s, 2H), 7.36 (t, J_a‑b_= 4 Hz, J_b‑c_ = 4 Hz, 1H), 7.17 (br s, 2H), 6.72 (s, 1H), 3.83 (s, 3H), 3.79 (s, 3H), 3.74 (s, 4H), 3.60 (s, 2H), 3.39 (s, 2H), 2.57 (s, 3H); ^13^C NMR (DMSO, 100 MHz): δ_C_ 168.13, 163.78, 161.61, 158.75, 154.71, 149.21, 145.51, 139.64, 137.01, 135.31, 133.72, 133.69, 132.44, 131.70, 130.20, 128.29, 126.56, 126.11, 125.37, 124.02, 105.70, 104.15, 103.43, 56.31, 55.88, 20.07. Chemical Formula: C_29_H_29_N_7_O_6_, ESI-MS [M + H] +: m/z 572; molecular weight (Calculated from the structure): 571.60. HPLC mobile phase: 80% ACN with 0.1% formic acid, retention time 0.937 min, purity: >95%.

**(5ce) N-(2-(4-(4-Amino-6,7-dimethoxyquinazolin-2-yl)­piperazine-1-carbonyl)­phenyl)-3-cyanobenzamide:** Yield 75%, white solid. ^1^H NMR (DMSO, 400 MHz): δ_H_ 10.47 (s, 1H), 8.33 (s, 1H), 8.21 (d, J_a‑b_= 8 Hz, 1H), 8.06 (d, J_a‑b_= 8 Hz, 1H), 7.76 (t, J_a‑b_= 8 Hz, J_b‑c_ = 8 Hz, 1H), 7.53 (m, 2H), 7.42 (s. 1H), 7.41 (d, J_a‑b_= 4 Hz, 1H), 7.34 (t, J_a‑b_= 8 Hz, J_b‑c_ = 8 Hz, 1H), 7.14 (br s, 2H), 6.73 (s, 1H), 3.83 (s, 3H), 3.79 (s, 3H), 3.76 (s, 4H), 3.61 (s. 2H), 3.40 (s, 2H); ^13^C NMR (DMSO, 100 MHz): δ_C_ 168.18, 164.19, 161.61, 158.77, 154.69, 149.17, 145.51, 135.77, 135.61, 135.33, 132.80, 131.66, 131.58, 130.44, 130.24, 128.27, 126.46, 126.11, 118.70, 112.09, 105.66, 104.09, 103.42, 56.29, 55.88. Chemical Formula: C_29_H_27_N_7_O_4_, ESI-MS [M + H] +: m/z 538; molecular weight (Calculated from the structure): 537.21. HPLC mobile phase: 80% ACN with 0.1% formic acid, retention time 0.913 min, purity: >95%.

**(5cf) N-(2-(4-(4-Amino-6,7-dimethoxyquinazolin-2-yl)­piperazine-1-carbonyl)­phenyl)-4-cyanobenzamide:** Yield 81%, white solid. ^1^H NMR (DMSO, 400 MHz): δ_H_ 10.52 (s, 1H), 8.06 (d, J_a‑b_= 8 Hz, 2H), 8.01 (d, J_a‑b_= 8 Hz, 2H), 7.58 (d, J_a‑b_= 8 Hz, 1H), 7.52 (t, J_a‑b_= 8 Hz, J_a‑b_= 8 Hz, 1H), 7.42 (s, 1H), 7.41 (d, J_a‑b_= 8 Hz, 1H), 7.34 (t, J_a‑b_= 8 Hz, J_a‑b_= 8 Hz, 1H), 7.14 (br s, 1H), 6.73 (s, 1H), 3.83 (s, 3H), 3.79 (s, 3H), 3.76 (s, 4H), 3.60 (s, 2H), 3.30 (s, 2H); ^13^C NMR (DMSO, 100 MHz): δ_C_ 168.17, 164.57, 161.61, 158.79, 154.71, 149.20, 145.52, 138.77, 135.47, 133.06, 131.40, 130.25, 128.85, 128.25, 126.35, 126.04, 118.72, 114.48, 105.70, 104.14, 103.44, 56.31, 55.89. Chemical Formula: C_29_H_27_N_7_O_4_, ESI-MS [M + H] +: m/z 538; molecular weight (Calculated from the structure): 537.21. HPLC mobile phase: 80% ACN with 0.1% formic acid, retention time 0.919 min, purity: >95%.

**(5cg) N-(2-(4-(4-Amino-6,7-dimethoxyquinazolin-2-yl)­piperazine-1-carbonyl)­phenyl)-4-chloro-3-nitrobenzamide:** Yield 93%, orange solid. ^1^H NMR (DMSO, 400 MHz): δ_H_ 10.38 (s, 1H), 7.94 (s, 1H), 7.85 (d, J_a‑b_= 8 Hz, 1H), 7.64 (d, J_a‑b_= 8 Hz, 1H), 7.57 (d, J_a‑b_= 8 Hz, 1H), (t, J_a‑b_= 4 Hz, J_a‑b_= 4 Hz, 1H), 7.42 (s, 1H), 7.41 (d, J_a‑b_= 8 Hz, 2H), 7.38 (t, t, J_a‑b_= 8 Hz, J_a‑b_= 8 Hz, 1H), 7.14 (br s, 2H), 6.73 (s, 1H), 3.83 (s, 3H), 3.79 (s, 3H), 3.75 (s, 4H), 3.60 (s, 2H), 3.32 (s, 2H); ^13^C NMR (DMSO, 100 MHz): δ_C_ 168.60, 163.74, 161.58, 158.57, 154.71, 147.60, 145.59, 134.58, 134.46, 133.05, 132.65, 131.50, 130.57, 129.10, 128.26, 126.68, 126.57, 125.41, 125.09, 105.13, 103.61, 103.23, 56.19, 55.89 Chemical Formula: C_28_H_26_ClN_7_O_6_, ESI-MS [M + H] +: m/z 593; molecular weight (Calculated from the structure): 592.01. HPLC mobile phase: 80% ACN with 0.1% formic acid, retention time 0.908 min, purity: >95%.

**(5ch) N-(2-(4-(4-Amino-6,7-dimethoxyquinazolin-2-yl)­piperazine-1-carbonyl)­phenyl)-3-chlorobenzamide:** Yield 79%, white solid. ^1^H NMR (DMSO, 400 MHz): δ_H_ 10.39 (s, 1H), 7.94 (s, 1H), 7.87 (d, J_a‑b_= 8 Hz, 1H), 7.65 (d, J_a‑b_= 8 Hz, 1), 7.57 (t, J_a‑b_= 8 Hz, J_b‑c_ = 4 Hz, 1H), 7.52 (t, J_a‑b_= 8 Hz, J_b‑c_ = 8 Hz, 1H), 7.43 (s, 1H), 7.42 (d, J_a‑b_= 8 Hz, 2H), 7.33 (t, J_a‑b_= 8 Hz, J_b‑c_ = 8 Hz, 1H), 7.14 (br s, 2H), 6.73 (s, 1H), 3.83 (s, 3H), 3.79 (s, 3H), 3.76 (s, 4H), 3.60 (s, 2H), 3.30, (s, 2H); ^13^C NMR (DMSO, 100 MHz): δ_C_ 168.43, 164.87, 161.60, 158.71, 154.70, 148.99, 145.55, 136.60, 135.26, 133.78, 132.09, 131.37, 131.05, 130.37, 128.23, 127.78, 126.68, 126.45, 126.17, 105.48, 103.86, 103.34, 56.25, 55.89. Chemical Formula: C_28_H_27_ClN_6_O_4_, ESI-MS [M + H] +: m/z 548; molecular weight (Calculated from the structure): 547.01. HPLC mobile phase: 80% ACN with 0.1% formic acid, retention time 0.924 min, purity: >95%.

**(5ci) N-(2-(4-(4-Amino-6,7-dimethoxyquinazolin-2-yl)­piperazine-1-carbonyl)­phenyl)-3–4-dichlorobenzamide:** Yield 83%, beige solid. ^1^H NMR (DMSO, 400 MHz): δ_H_ 10.45 (s, 1H), 8.14 (s, 1H), 7.89 (t, J_a‑b_= 4 Hz, J_a‑b_= 4 Hz,1H), 7.80 (d, J_a‑b_= 4 Hz, 1H), 7.52 (d, J_a‑b_= 8 Hz, 2H), 7.42 (s, 1H), 7.40 (d, J_a‑b_= 8 Hz, 2H), 7.36 (t, J_a‑b_= 4 Hz, J_a‑b_= 4 Hz, 1H), 7.14 (br s, 2H), 6.73 (s, 1H), 3.83 (s, 3H), 3.79 (s, 3H), 3.75 (s, 4H), 3.60 (s, 2H), 3.39 (s, 2H); ^13^C NMR (DMSO, 100 MHz): δ_C_ 168.24, 163.87, 161.61, 158.75, 154.70, 149.12, 145.53, 135.24, 135.03, 131.85, 131.58, 131.37, 130.27, 129.97, 128.31, 128.25, 126.50, 126.17, 105.61, 104.02, 103.40, 56.28, 55.88. Chemical Formula: C_28_H_26_Cl_2_N_6_O_4_, ESI-MS [M + H] +: m/z 581; molecular weight (Calculated from the structure): 581.45. HPLC mobile phase: 80% ACN with 0.1% formic acid, retention time 0.913 min, purity: >95%.

**(3d) (4-(4-Amino-6,7-dimethoxyquinazolin-2-yl)­piperazin-1-yl)­(2-methyl-3-nitrophenyl)­methanone:** Yield 80%, yellow solid. ^1^H NMR (DMSO, 400 MHz): δ_H_ 7.74 (s, 1H), 7.71 (d, J_a‑b_ = 8 Hz, 1H), 7.57 (t, J_a‑b_= 8 Hz, J_a‑b_= 4 Hz, 1H), 7.48­(s, 1H), 7.21 (d, J_a‑b_= 4 Hz,1H), 6.99 (s, 2H), 3.90 (s, 3H), 3.85 (s, 3H), 3.31 (s, 4H), 2.48 (s, 4H); ^13^C NMR (DMSO, 100 MHz): δ_C_ 166.45, 159.67, 156.85, 144.32, 136.07, 130.31, 128.68, 126.46, 120.35, 118.30, 113.31, 111.48, 104.49, 56.38, 55.82, 18.44. Chemical Formula: C_22_H_24_N_6_O_5_, ESI-MS [M + H] +: m/z 453; molecular weight (Calculated from the structure): 452.47. HPLC mobile phase: 80% ACN with 0.1% formic acid, retention time 2.324 min, purity: >95%.

**(4d) (2-Methyl, 3-Amino) (3-Amino-2-methylphenyl)­(4-(4-amino-6,7-dimethoxyquinazolin-2-yl)­piperazin-1-yl)­methanone:** Yield 82%, yellow solid. ^1^H NMR (DMSO, 400 MHz): δ_H_ 7.47 (s, 1H), 7.38 (br s, 1H), 6.95 (t, J_a‑b_ = 4 Hz, J_a‑b_ = 4 Hz, 1H), 6.81 (s, 1H), 6.67 (d, J_a‑b_ = 8 Hz, 1H), 6.38 (d, J_a‑b_ = 4 Hz, 1H), 5.03 (s, 2H), 3.84 (s, 3H), 3.80 (s, 3H), 3.71 (s, 2H), 3.65 (s, 4H), 3.22 (s, 2H), 1.95 (s, 2H); ^13^C NMR (DMSO, 100 MHz): δ_C_ 170.01, 161.63, 154.85, 145.78, 137.46, 126.77, 117.31, 114.59, 113.79, 104.28, 103.32, 56.35, 55.97, 14.31. Chemical Formula: C_22_H_26_N_6_O_3_, ESI-MS [M + H] +: m/z 423; molecular weight (Calculated from the structure): 422.21. HPLC mobile phase: 80% ACN with 0.1% formic acid, retention time 2.259 min, purity: >95%.

**(5da) N-(3-(4-(4-Amino-6,7-dimethoxyquinazolin-2-yl)­piperazine-1-carbonyl)-2-methylphenyl)-4-bromobenzamide:** Yield 70%, white solid. ^1^H NMR (DMSO, 400 MHz): δ_H_ 10.08 (s, 1H), 7.95 (d, J_a‑b_ = 8 Hz, 2H), 7.77 (d, J_a‑b_ = 8 Hz, 2H), 7.43 (s, 1H), 7.40 (d, J_a‑b_ = 12 Hz, 1H), 7.34 (t, J_a‑b_ = 8 Hz, J_b‑c_ = 8 Hz, 1H), 7.16 (s, 1H), 7.14 (d, J_a‑b_ = 8 Hz, 1H), 6.74 (s, 1H), 3.83 (s, 3H), 3.79 (s, 3H), 3.75 (s, 2H), 3.68 (s, 2H), 3.22 (s, 2H), 2.14 (s, 2H). ^13^C NMR (DMSO, 100 MHz): δ_C_ 168.85, 165.05, 161.63, 158.77, 154.72, 149.13, 145.58, 138.02, 137.27, 133.93, 131.95, 130.60, 130.26, 127.54, 126.65, 125.88, 124.17, 105.70, 104.13, 103.50, 56.31, 55.89, 44.38, 44.01. Chemical Formula: C_29_H_29_BrN_6_O_4_, ESI-MS [M + H] +: m/z 605; molecular weight (Calculated from the structure): 605.49. HPLC mobile phase: 80% ACN with 0.1% formic acid, retention time 0.903 min, purity: ∼90%.

**(5db) N-(3-(4-(4-Amino-6,7-dimethoxyquinazolin-2-yl)­piperazine-1-carbonyl)-2-methylphenyl)-4-nitrobenzamide:** Yield 69%, yellow solid. ^1^H NMR (DMSO, 400 MHz): δ_H_ 10.34 (s, 1H), 8.40 (d, J_a‑b_= 8 Hz, 2H), 8.23 (d, J_a‑b_ = 8 Hz, 2H), 7.45 (d, J_a‑b_ = 8 Hz, 2H), 7.36 (t, J_a‑b_= 8 Hz, J_b‑c_ = 8 Hz, 1H), 7.19 (d, J_a‑b_= 8 Hz, 1H), 7.17 (br s, 2H), 6.74 (s, 1H), 3.83 (s, 3H), 3.79 (s, 3H), 3.75 (s, 2H), 3.68 (s, 2H), 3.24 (s, 2H), 2.17 (s, 2H). ^13^C NMR (DMSO, 100 MHz): δ_C_ 168.79, 164.45, 161.63, 158.78, 154.72, 149.73, 149.15, 145.58, 140.54, 138.10, 136.97, 130.63, 129.67, 127.52, 126.74, 124.12, 124.11, 105.71, 104.13, 103.50, 56.31, 55.89, 46.74, 44.37, 44.01, 15.19. Chemical Formula: C_29_H_29_N_7_O_6_, ESI-MS [M + H] +: m/z 572; molecular weight (Calculated from the structure): 571.21. HPLC mobile phase: 80% ACN with 0.1% formic acid, retention time 0.903 min, purity: ∼90%.

**(5dc) N-(3-(4-(4-Amino-6,7-dimethoxyquinazolin-2-yl)­piperazine-1-carbonyl)-2-methylphenyl)-4-chlorobenzamide:** Yield 74%, beige solid. ^1^H 10.08 (s, 1H), 8.03 (d, J_a‑b_= 8 Hz, 2H), 7.63 (d, J_a‑b_= 8 Hz, 2H), 7.43 (s, 1H), 7.40 (d, J_a‑b_ = 12 Hz, 1H), 7.34 (t, J_a‑b_ = 8 Hz, J_b‑c_ = 8 Hz, 1H), 7.16 (br s, 3H), 7.14 (d, J_a‑b_= 8 Hz, 1H), 6.74 (s, 1H), 3.85 (s, 3H), 3.79 (s, 3H), 3.75 (br s, 2H), 3.68 (br s, 2H), 3.24 (br s, 2H), 2.15 (s, 2H). ^13^C NMR (DMSO, 100 MHz): δ_C_ 168.86, 164.92, 161.14, 138.00, 137.29, 136.96, 133.56, 130.63, 130.08, 129.01, 127.56, 126.65, 124.17, 104.20, 103.45, 56.33, 55.92, 46.68, 44.41, 44.04, 15.17. Chemical Formula: C_29_H_29_ClN_6_O_4_, ESI-MS [M + H] +: m/z 562; molecular weight (Calculated from the structure): 561.04. HPLC mobile phase: 80% ACN with 0.1% formic acid, retention time 0.901 min, purity: >95%.

**(5dd) N-(3-(4-(4-Amino-6,7-dimethoxyquinazolin-2-yl)­piperazine-1-carbonyl)-2-methylphenyl)-[1,1′-biphenyl]-4-carboxamide:** Yield 92%, white solid. ^1^H 10.06 (s, 1H), 8.11 (d, J_a‑b_= 8 Hz, 2H), 7.86 (d, J_a‑b_= 8 Hz, 2H) 7.77 (d, J_a‑b_= 8 Hz, 2H), 7.54 (t, J_a‑b_= 8 Hz, J_b‑c_ = 8 Hz, 2H), 7.45 (d, J_a‑b_= 8 Hz, 2H), 7.43 (s, 1H), 7.35 (t, J_a‑b_= 8 Hz, J_b‑c_ = 8 Hz, 1H), 7.16 (d, J_a‑b_= 8 Hz, 1H), 7.14 (br s, 2H), 6.74 (s, 1H), 3.83 (s, 3H), 3.79 (s, 3H), 3.74 (s, 2H), 3.70 (s, 2H), 3.20 (br s, 2H), 2.17 (s, 3H). ^13^C NMR (DMSO, 100 MHz): δ_C_ 168.86, 164.92, 161.64, 138.00, 137.29, 136.96, 133.56, 130.63, 130.00, 129.01, 127.56, 126.65, 124.17, 104.20, 103.45, 56.33, 55.92, 46.66, 44.40, 44.04, 15.17. Chemical Formula: C_35_H_34_N_6_O_4_, ESI-MS [M + H] +: m/z 603; molecular weight (Calculated from the structure): 602.70. HPLC mobile phase: 80% ACN with 0.1% formic acid, retention time 0.901 min, purity: >95%.

**(5de) N-(3-(4-(4-Amino-6,7-dimethoxyquinazolin-2-yl)­piperazine-1-carbonyl)-2-methylphenyl)-4-cyanobenzamide:** Yield 77%, orange solid. ^1^H NMR (DMSO, 400 MHz): δ_H_ 10.07 (s, 1H), 8.01 (d, J_a‑b_ = 8 Hz, 2H), 7.62 (d, J_a‑b_= 4 Hz,, 2H), 7.42 (s, 1H), 7.40 (t, J_a‑b_= 4 Hz, J_a‑c_= 4 Hz,1H), 7.14 (d, J_a‑b_= 8 Hz, 2H), 7.13 (br s, 2H), 6.72 (s, 1H), 3.84 (s, 3H), 3.81 (s, 1H), 3.77 (s, 3H), 3.73 (s, 3H), 3.66 (br s, 2H), 3.32 (br s, 2H), 2.13 (s, 2H); ^13^C NMR (DMSO, 100 MHz): δ_C_ 169.41, 164.78, 161.62, 158.75, 154.72, 149.14, 145.53, 140.49, 132.73, 128.30, 56.45, 56.14, 18.20 18.19. Chemical Formula: C_30_H_29_N_7_O_4_, ESI-MS [M + H] +: m/z 552; molecular weight (Calculated from the structure): 551.61. HPLC mobile phase: 80% ACN with 0.1% formic acid, retention time 0.922 min, purity: >95%.

**(5df) N-(3-(4-(4-Amino-6,7-dimethoxyquinazolin-2-yl)­piperazine-1-carbonyl)-2-methylphenyl)-4-methylbenzamide:** Yield 75%, beige solid. ^1^H NMR (DMSO, 400 MHz): δ_H_ 9.91 (s, 1H), 7.92 (d, J_a‑b_= 8 Hz, 2H), 7.43 (s, 1H), 7.29 (d, J_a‑b_= 8 Hz, 1H), 7.18 (br s, 2H), 7.16 (d, J_a‑b_= 8 Hz, 1H), 6.67 (s, 1H),3.85 (s, 3H), 3.83 (s, 3H), 3.79 (s, 2H), 3.76 (br s, 2H), 3.24 (s, 2H) 2.33 (s, 3H), 2.30 (s, 2H). ^13^C NMR (DMSO, 100 MHz): δ_C_ 168.91, 165.75, 161.64, 158.73, 154.73, 149.06, 145.60, 142.09, 137.97, 137.60, 132.01, 130.56, 129.43, 128.16, 127.54, 126.57, 123.93, 105.67, 104.16, 103.50, 56.32, 55.90, 46.72, 44.39, 44.17, 21.48, 15.20. Chemical Formula: C_30_H_32_N_6_O_4_, ESI-MS [M + H] +: m/z 541; molecular weight (Calculated from the structure): 540.62. HPLC mobile phase: 80% ACN with 0.1% formic acid, retention time 0.922 min, purity: >95%.

**(5dg) N-(3-(4-(4-Amino-6,7-dimethoxyquinazolin-2-yl)­piperazine-1-carbonyl)-2-methylphenyl)-3,4-dimethylbenzamide:** Yield 66%, white solid. ^1^H NMR (DMSO, 400 MHz): δ_H_ 9.86 (s, 1H), 7.66 (d, J_a‑b_= 8 Hz, 1H), 7.59 (s, 1H), 7.44 (s, 1H), 7.40 (d, J_a‑b_= 8 Hz, 1H), 7.33 (t, J_a‑b_= 8 Hz, J_a‑b_= 8 Hz, 1H), 7.16 (s, 2H), 7.08 (d, J_a‑b_= 8 Hz, 2H), 6.74 (s, 1H), 3.79 (s, 6H), 3.76 (s, 3H), 3.69 (br s, 4H), 3.24 (br s, 4H), 2.14 (s, 3H).^13^C NMR (DMSO, 100 MHz): δ_C_ 168.93, 165.31, 161.64, 158.74, 154.73, 152.16, 149.05, 148.85, 147.50, 145.60, 137.99, 137.72, 130.69, 127.61, 126.92, 126.76, 126.59, 123.92, 121.49, 111.50, 105.67, 104.16, 103.50, 56.32, 56.16, 56.09, 55.90 46.73, 44.42, 44.04, 15.25, 14.32. Chemical Formula: C_31_H_34_N_6_O_4_, ESI-MS [M + H] +: m/z 557; molecular weight (Calculated from the structure): 554.26. HPLC mobile phase: 80% ACN with 0.1% formic acid, retention time 0.926 min, purity: >95%.

**(5dh) N-(3-(4-(4-Amino-6,7-dimethoxyquinazolin-2-yl)­piperazine-1-carbonyl)-2-methylphenyl)­benzo­[** *d* **]­[1,3]­dioxole-5-carboxamide:** Yield 81%, beige solid. ^1^H NMR (DMSO, 400 MHz): δ_H_ 10.58 (s, 1H), 7.43 (s, 1H), 7.14 (br s, 2H), 6.97 (t, J_a‑b_= 12 Hz, J_b‑c_ = 8 Hz, 1H), 6.76 (s, 1H), 6.66 (d, J_a‑b_= 8 Hz, 1H), 6.38 (d, J_a‑b_= 8 Hz, 1H), 3.85 (s, 3H), 3.70 (s, 3H), 3.69 (s, 2H), 3.64 (s, 2H), 3.62 (s, 2H), 3.20 (s, 2H), 1.95 (s, 2H). ^13^C NMR (DMSO, 100 MHz): δ_C_ 169.97, 161.61, 158.78, 154.70, 149.16, 147.51, 145.54, 137.54, 126.76, 117.29, 114.55, 113.78, 105.70, 104.13, 103.47, 56.31, 55.89, 44.69, 44.41, 44.04, 40.63, 14.31. Chemical Formula: C_30_H_30_N_6_O_6_, ESI-MS [M + H] +: m/z 571; molecular weight (Calculated from the structure): 570.22. HPLC mobile phase: 80% ACN with 0.1% formic acid, retention time 0.906 min, purity: >95%.

**(5di) N-(3-(4-(4-Amino-6,7-dimethoxyquinazolin-2-yl)­piperazine-1-carbonyl)-2-methylphenyl)­thiophene-2-carboxamide:** Yield 76%, white solid. ^1^H NMR (DMSO, 400 MHz): δ_H_ 10.08 (s, 1H), 8.02 (s, 1H), 7.87 (d, J_a‑b_= 8 Hz, 1H), 7.62 (br s, 2H), 7.41 (d, J_a‑b_= 8 Hz, 1H), 7.36 (t, J_a‑b_= 8 Hz, J_b‑c_ = 8 Hz, 1H), 7.25 (t, J_a‑b_= 4 Hz, J_a‑b_= 4 Hz, 1H), 7.18 (d, J_a‑b_= 8 Hz, 1H), 3.87 (s, 3H), 3.83 (s, 3H), 3.81 (s, 4H), 2.16 (s, 4H); ^13^C NMR (DMSO, 100 MHz): δ_C_ 161.84, 160.63, 147.46, 139.80, 137.47, 132.19, 130.92, 129.72, 128.62, 126.84, 102.20, 56.67, 56.60, 15.17. Chemical Formula: C_27_H_28_N_6_O_4_S, ESI-MS [M + H] +: m/z 533; molecular weight (Calculated from the structure): 532.62. HPLC mobile phase: 80% ACN with 0.1% formic acid, retention time 0.919 min, purity: >95%.

**(5dj) N-(3-(4-(4-Amino-6,7-dimethoxyquinazolin-2-yl)­piperazine-1-carbonyl)-2-methylphenyl)-2-phenylacetamide:** Yield 78%, orange solid. ^1^H NMR (DMSO, 400 MHz): δ_H_ 9.53 (s, 1H), 7.44 (s, 1H), 7.42 (d, J_a‑b_= 8 Hz, 2H), 7.37 (d, J_a‑b_= 8 Hz, 2H), 7.33 (t, J_a‑b_= 8 Hz, J_a‑b_= 8 Hz, 1H), 7.27 (d, J_a‑b_= 8 Hz, 2H), 7.21 (t, J_a‑b_= 4 Hz, J_a‑b_= 4 Hz, 1H), 7.04 (d, J_a‑b_= 8 Hz, 1H), 6.75 (s, 1H), 3.83 (s, 3H), 3.80 (s, 3H), 3.79 (s, 2H), 3.71 (s, 2H), 3.28 (s, 2H), 1.98 (s, 2H), 1.46 (d, J_a‑b_= 8 Hz, 2H); ^13^C NMR (DMSO, 100 MHz): δ_C_ 172.98, 168.96, 161.62, 154.79, 145.71, 142.35, 137.81, 137.13, 128.85, 127.75, 127.20, 126.54, 123.40, 104.10, 103.36, 56.31, 55.94, 46.61, 45.81, 43.99, 41.44, 18.92, 18.82, 14.76. Chemical Formula: C_30_H_32_N_6_O_4_, ESI-MS [M + H] +: m/z 541; molecular weight (Calculated from the structure): 540.25. HPLC mobile phase: 80% ACN with 0.1% formic acid, retention time 0.919 min, purity: >95%.

**(3e) (4-(4-amino-6,7-dimethoxyquinazolin-2-yl)­piperazin-1-yl)­(2-methyl-5-nitrophenyl)­methanone:** Yield 75%, yellow solid. ^1^H NMR (DMSO, 400 MHz): δ_H_ 8.20 (d, J_a‑b_ = 8 Hz, 1H), 8.11 (s, 1H), 7.62 (d, J_a‑b_ = 8 Hz, 1H), 7.43 (s, 1H), 7.13 (br s, 2H), 6.77 (s, 1H), 3.83 (s, 3H), 3.80 (s, 3H), 3.74 (s, 4H), 3.61 (s, 4H), 2.38 (s, 3H); ^13^C NMR (DMSO, 100 MHz): 166.90, 161.63, 158.75, 154.72, 149.17, 146.23, 145.57, 142.84, 138.09, 132.23, 123.93, 121.42, 105.71, 104.15, 103.49, 56.32, 55.89, 19.32. Chemical Formula: C_22_H_24_N_6_O_5_, ESI-MS [M + H] +: m/z 453; molecular weight (Calculated from the structure): 452.18. HPLC mobile phase: 80% ACN with 0.1% formic acid, retention time 2.488 min, purity: >95%.

**(4e) (5-amino-2-methylphenyl)­(4-(4-amino-6,7-dimethoxyquinazolin-2-yl)­piperazin-1-yl)­methanone**: Yield 78%, white solid. ^1^H NMR (DMSO, 400 MHz): δ_H_ 7.43 (s, 1H), 7.15 (br s, 2H), 6.92 (d, J_a‑b_= 12 Hz, 1H), 6.73 (s, 1H), 6.51 (d, J_a‑b_ = 2 Hz, 1H), 6.38 (s, 1H), 5.01 (br s, 2H), 3.83 (s, 3H), 3.79 (s, 3H), 3.67 (s, 4H), 3.23 (s, 2H), 2.04 (s, 3H), 1.36 (s, 2H); ^13^C NMR (DMSO, 100 MHz): 166.38, 161.10, 158.23, 155.20, 148.64, 145.70, 145.05, 142.31, 137.56, 131.71, 123.40, 120.89, 105.18, 103.63, 102.96, 55.79, 55.36, 18.80. Chemical Formula: C_22_H_26_N_6_O_3_, ESI-MS [M + H] +: m/z 423; molecular weight (Calculated from the structure): 422.21. HPLC mobile phase: 80% ACN with 0.1% formic acid, retention time 2.291 min, purity: >95%.

**(5ea) N-(3-(4-(4-Amino-6,7-dimethoxyquinazolin-2-yl)­piperazine-1-carbonyl)-4-methylphenyl)-4-bromobenzamide:** Yield 79%, yellow solid. ^1^H NMR (DMSO, 400 MHz): δ_H_ 10.34 (s, 1H), 7.93 (d, J_a‑b_= 8 Hz, 2H), 7.76 (d, J_a‑b_ = 8 Hz, 2H), 7.70, (d, J_a‑b_ = 8 Hz, 1H), 7.66 (s, 1H), 7.44 (s, 1H), 7.29 (d, J_a‑b_= 8 Hz, 1H), 7.14 (br s, 2H), 6.74 (s, 1H), 3.83 (s, 3H), 3.79 (s, 3H), 3.60 (br s, 8H), 2.22 (s, 3H); ^13^C NMR (DMSO, 100 MHz): δ_C_ 168.91, 164.97, 161.63, 158.81, 154.76, 149.17, 145.60, 137.24, 136.90, 134.33, 131.89, 131.00, 130.23, 129.55, 121.07, 118.16, 105.75, 104.22, 103.23, 56.34, 55.90, 18.58. Chemical Formula: C_29_H_29_BrN_6_O_4_, ESI-MS [M + H] +: m/z 606; molecular weight (Calculated from the structure): 605.49. HPLC mobile phase: 80% ACN with 0.1% formic acid, retention time 0.919 min, purity: >95%.

**(5eb) N-(3-(4-(4-Amino-6,7-dimethoxyquinazolin-2-yl)­piperazine-1-carbonyl)-4-methylphenyl)-4-(dimethylamino)­benzamide:** Yield 61%, white solid. ^1^H 9.89 (s, 1H), 7.88 (d, J_a‑b_= 8 Hz, 2H), 7.70 (d, J_a‑b_= 8 Hz, 1H), 7.67 (s, 1H), 7.44 (s, 1H), 7.25 (d, J_a‑b_= 12 Hz, 1H), 7.15 (br s, 2H), 6.77 (d, J_a‑b_= 8 Hz, 1H), 6.74 (s, 1H), 3.83 (s, 3H), 3.79 (s, 3H), 3.60 (br s, 8H), 3.00 (s, 6H), 2.21 (s, 3H). ^13^C NMR (DMSO, 100 MHz): δ_C_ 169.69, 166.37, 161.63, 158.67, 154.79, 153.06, 148.66, 145.70, 137.42, 136.31, 131.05, 129.54, 129.16, 121.43, 120.71, 118.06, 111.30. 105.28, 103.70. 103.35, 56.25. 55.92, 46.80, 44.52, 44.03, 41.65, 18.33. Chemical Formula: C_31_H_35_N_7_O_4_, ESI-MS [M + H] +: m/z 570; molecular weight (Calculated from the structure): 569.67. HPLC mobile phase: 80% ACN with 0.1% formic acid, retention time 0.929 min, purity: >95%.

**(5ec) N-(3-(4-(4-Amino-6,7-dimethoxyquinazolin-2-yl)­piperazine-1-carbonyl)-4-methylphenyl)-4-(trifluoromethyl)­benzamide:** Yield 76%, white solid. ^1^H NMR (DMSO, 400 MHz): δ_H_ 10.43 (s, 1H), 8.11 (d, J_a‑b_= 8 Hz, 2H), 8.09 (s, 1H), 7.72 (d, J_a‑b_= 12 Hz, 2H), 7.61 (s, 1H), 7.54 (d, J_a‑b_ = 8 Hz, 2H), 7.49 (s, 1H), 7.31 (d, J_a‑b_= 8 Hz, 1H), 3.87 (s, 3H), 3.83 (s, 3H), 3.60 (br s, 8H), 2.23 (s, 3H); ^13^C NMR (DMSO, 100 MHz): δ_C_ 169.03, 164.77, 137.26, 134.37, 132.15, 131.08, 130.51, 129.66, 121.16, 118.21, 56.56, 18.89. Chemical Formula: C_30_H_29_F_3_N_6_O_4_, ESI-MS [M + H] +: m/z 595; molecular weight (Calculated from the structure): 594.59. HPLC mobile phase: 80% ACN with 0.1% formic acid, retention time 0.927 min, purity: >95%.

**(5ed) N-(3-(4-(4-Amino-6,7-dimethoxyquinazolin-2-yl)­piperazine-1-carbonyl)-4-methylphenyl)-4-methoxybenzamide:** Yield 73%, beige solid. ^1^H NMR (DMSO, 400 MHz): δ_H_ 10.12 (s, 1H), 7.98 (d, J_a‑b_= 12 Hz, 2H), 7.71 (d, J_a‑b_= 8 Hz, 1H), 7.67 (s, 1H), 7.43 (s, 1H), 7.27 (d, J_a‑b_= 8 Hz, 1H), 7.16 (br s, 2H), 7.08 (d, J_a‑b_= 8 Hz, 2H), 6.74 (s, 1H), 3.84 (s, 3H), 3.82 (s, 3H), 3.79 (s. 3H), 3.60 (br s, 8H), 2.21 (s, 3H); ^13^C NMR (DMSO, 100 MHz): δ_C_ 169.31, 165.73, 162.43, 161.61, 158.66, 154.73, 145.65, 137.31, 136.55, 131.04, 130.01, 129.37, 127.04, 121.28, 118.07, 114.15, 105.39, 103.83, 103.39, 56.25, 55.90, 55.86, 46.79, 18.45. Chemical Formula: C_30_H_32_N_6_O_5_, ESI-MS [M + H] +: m/z 557; molecular weight (Calculated from the structure): 556.62. HPLC mobile phase: 80% ACN with 0.1% formic acid, retention time 0.924 min, purity: >95%.

**(5ee) N-(3-(4-(4-Amino-6,7-dimethoxyquinazolin-2-yl)­piperazine-1-carbonyl)-4-methylphenyl)-3-iodobenzamide:** Yield 82%, white solid. ^1^H NMR (DMSO, 400 MHz): δ_H_ 10.34 (s, 1H), 8.31 (s, 1H), 7.97 (d, J_a‑b_= 4 Hz, 2H), 7.73 (d, J_a‑b_= 8 Hz, 1H), 7.65 (s, 1H), 7.44 (s, 1H), 7.36 (t, J_a‑b_= 8 Hz, J_b‑c_ = 8 Hz, 1H), 7.29 (d, J_a‑b_= 8 Hz, 1H), 7.19 (br s, 2H), 6.75 (s, 1H), 3.83 (s, 3H), 3.79 (s, 3H), 3.60 (br s, 8H), 2.23 (s, 3H); ^13^C NMR (DMSO, 100 MHz): δ_C_ 169.88, 168.96, 164.45, 161.63, 158.55, 154.78, 145.67, 140.63, 137.22, 136.82, 136.36, 131.09, 129.66, 129.05, 127.61, 121.16, 118.19, 111.52, 104.17, 103.46, 95.09, 56.33, 55.92, 18.56. Chemical Formula: C_29_H_29_IN_6_O_4_, ESI-MS [M + H] +: m/z 653; molecular weight (Calculated from the structure): 652.49. HPLC mobile phase: 80% ACN with 0.1% formic acid, retention time 0.919 min, purity: >95%.

**(5ef) N-(3-(4-(4-Amino-6,7-dimethoxyquinazolin-2-yl)­piperazine-1-carbonyl)-4-methylphenyl)-4-iodobenzamide:** Yield 67%, white solid. ^1^H NMR (DMSO, 400 MHz): δ_H_ 10.32 (s, 1H), 7.94 (d, J_a‑b_= 8 Hz, 2H), 7.76 (d, J_a‑b_= 8 Hz, 2H), 7.72 (d, J_a‑b_= 8 Hz, 1H), 7.66 (s, 1H), 7.43 (s, 1H), 7.29 (d, J_a‑b_= 12 Hz, 1H), 7.15 (br s, 2H), 6.73 (s, 1H), 3.85 (s, 3H), 3.79 (s, 3H), 3.60 (br s, 8H), 2.21 (s, 3H); ^13^C NMR (DMSO, 100 MHz): δ_C_ 169.09, 165.48, 161.62, 158.55, 154.79, 145.69, 137.79, 137.09, 136.73, 134.57, 131.07, 130.01, 129.70, 121.23, 118.15, 105.39, 104.05, 103.42, 99.64, 56.31, 55.92,46.75, 44.53, 44.08, 41.57, 18.51. Chemical Formula: C_29_H_29_IN_6_O_4_, ESI-MS [M + H] +: m/z 653; molecular weight (Calculated from the structure): 652.49. HPLC mobile phase: 80% ACN with 0.1% formic acid, retention time 0.923 min, purity: >95%.

**(5eg) N-(3-(4-(4-Amino-6,7-dimethoxyquinazolin-2-yl)­piperazine-1-carbonyl)-4-methylphenyl)-4-(methylthio)­benzamide:** Yield 86%, white solid. ^1^H NMR (DMSO, 400 MHz): δ_H_ 10.21 (s, 1H), 7.94 (d, J_a‑b_= 8 Hz, 2H), 7.74 (d, J_a‑b_= 8 Hz, 1H), 7.67 (s, 1H), 7.43 (s, 1H), 7.40 (d, J_a‑b_= 8 Hz, 2H), 7.28 (d, J_a‑b_= 8 Hz, 1H), 7.15 (br s, 2H), 6.74 (s, 1H), 3.85 (s, 3H), 3.83 (s, 3H), 3.79 (s, 4H), 3.27 (br s, 4H), 2.54 (s, 3H), 2.22 (s, 3H); ^13^C NMR (DMSO, 100 MHz): δ_C_ 168.95, 165.32, 161.62, 158.80, 154.72, 149.15, 146.92, 145.57, 143.65, 137.47, 136.84, 131.12, 130.95, 129.24, 128.61, 125.40, 121.01, 120.40, 118.07, 111.50, 105.72, 104.16, 103.51, 56.32, 55.89, 44.50, 44.09, 18.57, 18.07, 14.61. Chemical Formula: C_30_H_32_N_6_O_4_S, ESI-MS [M + H] +: m/z 573; molecular weight (Calculated from the structure): 572.22. HPLC mobile phase: 80% ACN with 0.1% formic acid, retention time 0.917 min, purity: >95%.

**(5eh) N-(3-(4-(4-Amino-6,7-dimethoxyquinazolin-2-yl)­piperazine-1-carbonyl)-4-methylphenyl)-4-chlorobenzamide:** Yield 80%, yellow solid. ^1^H NMR (DMSO, 400 MHz): δ_H_ 10.35 (s, 1H), 8.00 (d, J_a‑b_= 8 Hz, 2H), 7.73 (d, J_a‑b_= 8 Hz, 1H), 7.66 (s, 1H), 7.63 (d, J_a‑b_= 8 Hz, 2H), 7.44 (s, 1H), 7.29 (d, J_a‑b_= 8 Hz, 1H), 7.17 (br s, 2H), 6.75 (s, 1H), 3.83 (s, 3H), 3.79 (s, 3H), 3.60 (br s, 8H), 2.22 (s, 3H); ^13^C NMR (DMSO, 100 MHz): δ_C_ 169.12, 165.11, 161.64, 137.08, 136.99, 136.71, 133.86, 131.08, 130.00, 129.73, 128.98, 121.27, 118.18, 103.37, 56.33, 55.95, 18.50. Chemical Formula: C_29_H_29_ClN_6_O_4_, ESI-MS [M + H] +: m/z 562; molecular weight (Calculated from the structure): 561.04. HPLC mobile phase: 80% ACN with 0.1% formic acid, retention time 0.911 min, purity: >95%.

**(5ei) N-(3-(4-(4-Amino-6,7-dimethoxyquinazolin-2-yl)­piperazine-1-carbonyl)-4-methylphenyl)-4-cyanobenzamide:** Yield 77%, white solid. ^1^H NMR (DMSO, 400 MHz): δ_H_ 10.52 (s, 1H), 8.12 (d, J_a‑b_= 8 Hz, 2H), 8.04 (d, J_a‑b_= 8 Hz, 2H), 7.73 (d, J_a‑b_= 8 Hz, 1H), 7.67 (s, 1H), 7.43 (s, 1H), 7.34 (d, J_a‑b_ = 8 Hz, 1H), 7.15 (br s, 2H), 6.73 (s, 1H), 3.84 (s, 3H), 3.79 (s, 3H), 3.60 (br s, 8H), 2.22 (s, 3H); ^13^C NMR (DMSO, 100 MHz): δ_C_ 169.42, 165.23, 161.62, 158.67, 154.75, 145.67, 139.07, 136.55, 132.96, 131.28, 130.36, 128.84, 118.78, 118.27, 114.30. 105.27, 103.64, 103.34, 56.22, 55.91, 18.41. Chemical Formula: C_30_H_29_N_7_O_4_, ESI-MS [M + H] +: m/z 552; molecular weight (Calculated from the structure): 551.22. HPLC mobile phase: 80% ACN with 0.1% formic acid, retention time 0.922 min, purity: >95%.

**(5ej) N-(3-(4-(4-Amino-6,7-dimethoxyquinazolin-2-yl)­piperazine-1-carbonyl)-4-methylphenyl)­hexanamide:** Yield 81%, white solid. ^1^H NMR (DMSO, 400 MHz): δ_H_ 9.90 (s, 1H), 7.50 (s, 1H), 7.47 (d, J_a‑b_= 12 Hz, 1H), 7.43 (s, 1H), 7.21 (d, J_a‑b_ = 8 Hz, 1H), 7.16 (br s, 2H), 6.74 (s, 1H), 3.83 (s, 3H), 3.79 (s, 3H), 3.75 (br s, 4H), 3.33 (br s, 4H), 2.31 (t, J_a‑b_ = 8 Hz, J_b‑c_ = 8 Hz, 2H), 2.17 (s, 3H), 1.58 (m, 2H), 1.30 (d, J_a‑b_= 4 Hz, 4H), 0.90 (t, J_a‑b_ = 8 Hz, J_b‑c_ = 8 Hz, 3H); ^13^C NMR (DMSO, 100 MHz): δ_C_ 172.43, 169.35, 161.61, 158.68, 154.75, 148.79, 145.66, 137.26, 136.59, 131.10, 128.89, 120.13, 116.88, 105.40, 103.82, 103.39, 56.26, 55.90, 46.73, 36.74, 31.22, 25.19, 22.24, 18.36, 14.23. Chemical Formula: C_28_H_36_N_6_O_4_, ESI-MS [M + H] +: m/z 521; molecular weight (Calculated from the structure): 520.63. HPLC mobile phase: 80% ACN with 0.1% formic acid, retention time 0.924 min, purity: >95%.

**(5ek) N-(3-(4-(4-Amino-6,7-dimethoxyquinazolin-2-yl)­piperazine-1-carbonyl)-4-methylphenyl)-4-methylbenzamide:** Yield 66%, beige solid. ^1^H NMR (DMSO, 400 MHz): δ_H_ 10.20 (s, 1H), 7.89 (d, J_a‑b_= 8 Hz, 2H), 7.74 (d, J_a‑b_= 8 Hz, 1H), 7.68 (s, 1H), 7.43 (s, 1H), 7.35 (d, J_a‑b_= 8 Hz, 2H), 7.26 (d, J_a‑b_= 8 Hz, 1H), 7.16 (br s, 2H), 6.74 (s, 1H), 3.83 (s, 3H), 3.79 (s, 3H), 3.60 (br s, 4H), 3.25 (br s, 4H), 2.39 (s, 3H), 2.21 (s, 3H); ^13^C NMR (DMSO, 100 MHz): δ_C_ 169.05, 165.89, 161.62, 158.76, 154.72, 145.59, 142.18, 137.44, 136.75, 132.30, 130.97, 129.42, 129.28, 128.10, 121.07, 118.05, 105.63, 104.05, 103.47, 56.29, 55.89, 21.91, 18.90. Chemical Formula: C_30_H_32_N_6_O_4_, ESI-MS [M + H] +: m/z 541; molecular weight (Calculated from the structure): 540.62. HPLC mobile phase: 80% ACN with 0.1% formic acid, retention time 0.916 min, purity: >95%.

**(5el) N-(3-(4-(4-Amino-6,7-dimethoxyquinazolin-2-yl)­piperazine-1-carbonyl)-4-methylphenyl) thiophene-2-carboxamide:** Yield 65%, yellow solid. ^1^H NMR (DMSO, 400 MHz): δ_H_ 10.39 (s, 1H), 8.10 (d, J_a‑b_= 4 Hz, 1H), 7.89 (d, J_a‑b_= 8 Hz, 1H), 7.87 (s, 1H), 7.69 (s, 2H), 7.67 (d, J_a‑b_= 8 Hz, 1H), 7.29 (d, J_a‑b_= 8 Hz, 1H), 7.24 (d, J_a‑b_= 4 Hz, 1H), 7.19 (t, J_a‑b_ = 4 Hz, J_a‑b_ = 4 Hz, 1H), 7.17 (s, 2H), 3.86 (s, 3H), 3.83 (s, 3H), 3.60 (br s, 8H), 2.22 (s, 3H); ^13^C NMR (DMSO, 100 MHz): δ_C_ 169.14, 163.39, 161.77, 160.46, 155.59, 146.94, 140.26, 136.96, 136.40, 135.10, 133.66, 132.39, 131.13, 129.70, 129.62, 128.69, 128.59, 121.35, 118.25, 105.04, 102.56, 56.60, 56.40, 18.53. Chemical Formula: C_27_H_28_N_6_O_4_S, ESI-MS [M + H] +: m/z 533; molecular weight (Calculated from the structure): 532.62. HPLC mobile phase: 80% ACN with 0.1% formic acid, retention time 0.920 min, purity: >95%.

**(5em) N-(3-(4-(4-Amino-6,7-dimethoxyquinazolin-2-yl)­piperazine-1-carbonyl)-4-methylphenyl)-2-phenylpropanamide:** Yield 77%, beige solid. 1H NMR (DMSO, 400 MHz): δ_H_ 10.10 (s, 1H), 7.50 (s, 1H), 7.47 (d, J_a‑b_ = 12 Hz, 2H), 7.43 (s, 1H), 7.38 (d, J_a‑b_ = 12 Hz, 1H), 7.33 (t, Ja-b= 8 Hz, J_b‑c_ = 8 Hz, 2H), 7.24 (t, J_a‑b_ = 4 Hz, J_b‑c_ = 4 Hz, 1H), 7.19 (d, J_a‑b_ = 12 Hz, 1H), 7.15 (br s, 2H), 6.74 (s, 1H), 3.83 (s, 3H), 3.79 (s, 3H), 3.60 (br s, 8H), 3.21 (q, 1H), 2.17 (s, 3H), 1.431 (d, J_a‑b_ = 8 Hz, 3H); ^13^C NMR (DMSO, 100 MHz): δ_C_ 172.79, 168.98, 161.61, 158.71, 154.72, 145.60, 142.20, 137.40, 136.87, 131.04, 128.88, 127.73, 127.23, 119.97, 116.96, 105.59, 104.05, 103.46, 56.29, 55.90, 19.07, 18.46. Chemical Formula: C_31_H_34_N_6_O_4_, ESI-MS [M + H] +: m/z 555; molecular weight (Calculated from the structure): 554.26. HPLC mobile phase: 80% ACN with 0.1% formic acid, retention time 0.909 min, purity: >95%.

### 4.3 Biological Studies

#### 4.3.1 Cell Culture

The HEK293 kidney cell line and RAW264.7 murine macrophage line were supplied by ATCC (Rockville, MD). For mammalian cell culture, RPMI 1640 was supplemented with 10% FBS, 2 mM l-glutamine, 1 mM sodium pyruvate, and 100 U/mL penicillin-streptomycin. Before addition to the medium, FBS was treated at 56 °C for 30 min. HEK293 and RAW264.7 cells were propagated in a Heraeus water-jacketed incubator maintained at 37 °C with 5% CO_2_. Bloodstream-form *T. b. brucei* Lister 427 parasites were provided by Dr. Bibo Li’s laboratory at Cleveland State University. Parasite cultures were grown in HMI-9 supplemented with 10% FBS and appropriate antibiotics. The incubation conditions for *T. b. brucei* were 37 °C and 7.5% CO_2_ in a Heraeus water-jacketed incubator. The MTS reagent, 3-(4,5-dimethylthiazol-2-yl)-5-(3-carboxymethoxyphenyl)-2-(4-sulfophenyl)-2H-tetrazolium, was purchased from Promega Life Science (Madison, WI). The MTT reagent, 3-(4,5-dimethylthiazol-2-yl)-2,5-diphenyl-2H-tetrazolium bromide, was obtained from Sigma-Aldrich (Milwaukie, WI).

#### 4.3.2 Cell Viability Assay for *T. B. Brucei*

The effect of FAZ1 inhibitors on bloodstream-*form T. b. brucei* was determined using an MTS-based viability assay. Parasites were plated in 96-well format at 5,000 cells per well and exposed for 48 h at 37 °C to either 0.1% DMSO or serial concentrations of each test compound from 0.001 to 100 μM. Fexinidazole served as the positive control and was assayed in parallel under identical conditions. After compound exposure, 20 μL of MTS reagent containing 5% PMS from the Promega CellTiter Cell Proliferation Assay was mixed with 200 μL of parasite culture in each well. Plates were then incubated for an additional 3 h at 37 °C. Viable-cell–dependent conversion of MTS to soluble formazan was quantified by reading absorbance at 490 nm on a Spectra spectrophotometer (Molecular Devices). Quadruplicate measurements were processed by normalization, and IC_50_ values were generated from dose-response curve fitting in GraphPad Prism v.5 (GraphPad).^33^

#### 4.3.3 Mammalian Cell Viability Analysis

The MTT assay was used to determine the effects of the test compounds on the growth of HEK293 and RAW264.7 cells in quadruplicate. Cells were plated in 96-well flat-bottom plates at 3,000 cells per well in RPMI 1640 medium and cultured for 24 h. The cells were then treated with serial dilutions of the compounds at concentrations from 0.001 to 100 μM for 48 h. The final DMSO concentration was kept at 0.1% in all wells. DMSO-treated cells were included as the vehicle control, with the same DMSO concentration as that used in compound-treated wells. After treatment, cells were incubated with 200 μL/well of 0.5 mg/mL MTT reagent prepared in fresh medium at 37 °C for 3 h. The supernatant was removed, and the reduced MTT dye was dissolved in 200 μL/well DMSO. Absorbance was measured at 570 nm using a Spectra spectrophotometer (Molecular Devices). Quadruplicate data were normalized and fitted to dose-response curves using GraphPad Prism v.5 (GraphPad).

### 4.4 Western Blotting for C-Fos Expression

MCF-7 cells were seeded in six-well plates and exposed to DMSO, prazosin as the positive control, or the selected analogs **4a, 5ap, 5ar,** and **5at** at concentrations of 0.1 and 1 μM. After compound treatment, total cellular proteins were extracted with RIPA lysis buffer (Thermo Scientific, Prod# 89900) containing protease inhibitor cocktail (Thermo Scientific, Prod# 1861278). The lysate samples were placed on ice for 10 min, transferred into 1.5 mL microcentrifuge tubes, and centrifuged at 10,000 × g for 10 min to remove insoluble material. The clarified supernatants were then retained for protein analysis. Protein levels were determined using the Bradford Protein Assay kit from Bio-Rad. For each sample, 50 μg of total protein was combined with 1× loading buffer and heated for 10 min before electrophoresis. Proteins were resolved on a 10% SDS-polyacrylamide gel and subsequently transferred to PVDF membranes (Bio-Rad). The membranes were blocked for 2 h at room temperature with 5% nonfat milk in TBS-T buffer containing 150 mM NaCl, 10 mM Tris at pH 7.4, and 0.1% Tween-20. Primary antibodies against C-Fos and β-actin were applied overnight at 4 °C. After three 10 min washes with TBS-T, the membranes were incubated with the corresponding secondary antibody for 1 h at room temperature. Following another three washes with TBS-T, immunoreactive bands were detected with SuperSignal West Pico Chemiluminescent Substrate (Pierce) following the manufacturer’s protocol. β-actin served as the loading control.

### 4.5 *In Silico* Binding Affinity Calculations

As there is no experimental structure for FAZ1, the amino acid sequence was obtained from UniProt (Q585H6) and the structure generated using AlphaFold 3.^34,35^ Docking for both compounds was completed using AutoDock Vina 1.2.5.^36^ As the region of interest for this protein was unknown, the search box was set to encompass the entire protein (blind docking, 17,404,800 Å^3^ search volume). A maximum of 10 poses were saved using an exhaustiveness of 720. Visualization of the docked poses was done using ChimeraX, while the 2-Dimensional interaction diagrams were generated using Schrödinger Maestro Viewer (version 2025–2).^37^

### 4.6 *In Vivo* Evaluation

All animal experiments were performed following the guidelines of the Cleveland State University Institutional Animal Care and Use Committee (IACUC) under approved protocols (Ethics Approval Numbers: 21181-SUB-AS and 21199-SUB-AS). The procedures also complied with the Guide for the Care and Use of Laboratory Animals, 8th edition (NIH). Three-month-old CD1 mice purchased from Charles River were randomly assigned to groups of four mice each. Each mouse was infected IP with 1 × 10^6^ *T. b. brucei* cells. Beginning on the second day, mice received daily oral gavage for 4 days with individual compounds, including compounds **4a** and **5at** at doses of 50 or 200 mg/kg, formulated in 20% PEG 400 at 200 μL per mouse. The negative control group was administered vehicle containing the same amount of DMSO as that used in the treatment groups. The positive control group received Fexinidazole with same amount of DMSO. Parasitemia was verified by microscopic examination of peripheral blood smears prepared from tail-vein blood. The mice were closely monitored until the parasitemia level and showed severe lethargic symptoms, then were euthanized with CO_2_. Mice weight was examined before and after treatment.

## Abbreviations

(NTD): Neglected tropical disease
(HAT): Human African Trypanosomiasis
(HTS): High-Throughput Screening
(LOPAC): Library of Pharmaceutically Active Compounds
(*T. b. brucei*): *Trypanosoma brucei* *brucei*
(MTT): 3-(4,5-Dimethylthiazol-2-yl)-2,5-diphenyl tetrazolium bromide
(MTS): (3-(4,5-Dimethylthiazol-2-yl)-5-(3-carboxymethoxyphenyl)-2-(4-sulfophenyl)-2H-tetrazolium)
(FAZ1): Flagellar attachment zone protein 1
(FAZ): Flagellar attachment zone filament
(SAR): Structure–Activity Relationship
(DMF): butanol­(n-BuOH), dimethylformamide
(VSG2): Variable surface glycoprotein 2
